# Patterns of Genetic Variation in a Soybean Germplasm Collection as Characterized with Genotyping-by-Sequencing

**DOI:** 10.3390/plants10081611

**Published:** 2021-08-05

**Authors:** Yong-Bi Fu, Elroy R. Cober, Malcolm J. Morrison, Frédéric Marsolais, Gregory W. Peterson, Carolee Horbach

**Affiliations:** 1 Plant Gene Resources of Canada, Saskatoon Research and Development Centre, Agriculture and Agri-Food Canada, 107 Science Place, Saskatoon, SK S7N 0X2, Canada; gregory.peterson@agr.gc.ca (G.W.P.); carolee.horbach@agr.gc.ca (C.H.); 2 Ottawa Research and Development Centre, Agriculture and Agri-Food Canada, Ottawa, ON K1A 0C6, Canada; elroy.cober@agr.gc.ca (E.R.C.); malcolm.morrison@agr.gc.ca (M.J.M.); 3 Genomics and Biotechnology, London Research and Development Centre, Agriculture and Agri-Food Canada, London, ON N5V 4T3, Canada; frederic.marsolais@agr.gc.ca

**Keywords:** soybean, genetic distinctness, genetic redundancy, accession duplication, genomic characterization, genotyping-by-sequencing

## Abstract

Genomic characterization is playing an increasing role in plant germplasm conservation and utilization, as it can provide higher resolution with genome-wide SNP markers than before to identify and analyze genetic variation. A genotyping-by-sequencing technique was applied to genotype 541 soybean accessions conserved at Plant Gene Resources of Canada and 30 soybean cultivars and breeding lines developed by the Ottawa soybean breeding program of Agriculture and Agri-Food Canada. The sequencing generated an average of 952,074 raw sequence reads per sample. SNP calling identified 43,891 SNPs across 20 soybean chromosomes and 69 scaffolds with variable levels of missing values. Based on 19,898 SNPs with up to 50% missing values, three distinct genetic groups were found in the assayed samples. These groups were a mixture of the samples that originated from different countries and the samples of known maturity groups. The samples that originated from Canada were clustered into all three distinct groups, but 30 Ottawa breeding lines fell into two groups only. Based on the average pairwise dissimilarity estimates, 40 samples with the most genetic distinctness were identified from three genetic groups with diverse sample origin and known maturity. Additionally, 40 samples with the highest genetic redundancy were detected and they consisted of different sample origins and maturity groups, largely from one genetic group. Moreover, some genetically duplicated samples were identified, but the overall level of genetic duplication was relatively low in the collection. These findings are useful for soybean germplasm management and utilization.

## 1. Introduction

Genomic characterization of ex situ plant germplasm is playing an increasingly important role in germplasm management and utilization [1,2,3,4]. Characterizing germplasm using genomic tools has been technically and practically more feasible than before, thanks to the recent development of next generation sequencing technologies and bioinformatics tools, and drastically decreasing costs [5]. Such characterization can provide high resolution with genome-wide SNP markers to analyze genetic diversity and structure (e.g., see Milner et al. [3]; Sansaloni et al. [4]), group germplasm for the development of core collections (e.g., Jeong et al. [6]), and can support identification of genetic duplicates (e.g., see Ellis et al. [7]; Singh et al. [8]) for better germplasm management. It can also enhance the search for unique germplasm with traits of breeding targets for better varietal development (e.g., see Sansaloni et al. [4]; Mascher et al. [9]).

Considerable efforts were made using genomic tools to characterize ex situ soybean [*Glycine* spp.] germplasm (e.g., see Jeong et al. [6]; Song et al. [10]) and to develop different core subsets of soybean accessions in different genebanks (e.g., see Wang et al. [11]; Cho et al. [12]; Oliveira et al. [13]; Kaga et al. [14]; Priolli et al. [15]). These characterizations not only allow for a better understanding of the genetic variation present in soybean germplasm, but also support enhanced soybean germplasm management and utilization. For example, Song et al. [10] reported considerable redundant germplasm in the USDA soybean collection with 23% of *G. max* accessions and 30% of *G. soja* accessions being at least 99.9% identical, based on 42,509 SNPs, to another accession in the collection. Moellers et al. [16] demonstrated the effectiveness of the USDA soybean core collection in the identification of multiple soybean *Sclerotinia sclerotiorum* resistances. However, some challenges remain in the genomic characterization of all 176,000 soybean accessions that are currently conserved in more than 24 genebanks worldwide [17,18]. Little is known about the extent of genetic diversity and unique accessions in each collection.

Plant Gene Resources of Canada (PGRC; the Canadian national seed genebank at Saskatoon) maintains a soybean germplasm collection of 1031 accessions. These accessions were acquired largely from Canadian soybean breeding programs over the last 50 years and from the USDA-ARS soybean collection and N.I. Vavilov All-Russian Institute of Plant Genetic Resources for accessions with known early maturity over the last 15 years. Little is known about the genetic diversity and structure of the PGRC collection. Thus, a genomic characterization of the collection was initiated in 2017 with the objective of collecting information to enhance soybean germplasm management and utilization. To make the characterization more relevant to soybean germplasm utilization, we also included 30 soybean cultivars and breeding lines developed and released from the Ottawa soybean breeding program of Agriculture and Agri-Food Canada (AAFC). The specific objectives of the characterization were to: (1) apply a genotyping-by-sequencing technique to genotype 541 PGRC soybean accessions and 30 Ottawa breeding lines; (2) analyze the patterns of genetic diversity in the assayed samples; and (3) identify accessions with the most genetic distinctness and redundancy.

## 2. Materials and Methods

### 2.1. Soybean Germplasm and DNA Extraction

The soybean collection conserved at PGRC has 1031 accessions, but only 743 accessions were available for seed distribution in 2016. For this study, we selected 541 accessions from the 743 accessions representing 28 countries of origin and one group of unknown origin, based on the log proportion of the group size with respect to the 29 groups involved. Fortunately, the selection also included 269 accessions with three known maturity groups (MG) (38 for MG 0, 172 for MG 00 and 59 for MG 000) [19]. Note that soybean cultivars are commonly classified into 13 MGs, depending on their adaptation to photoperiod and seasonal temperature. In Canada, the earliest soybean cultivars are MG 000, which are adapted to a region north of 50° N. MG 00 and MG 0 cultivars are adapted to Manitoba, Eastern and Central Ontario and Western Quebec, while MG I and later are adapted to regions in Southern Ontario and Southern Quebec. To enhance our analysis of early maturity germplasm, we also acquired 30 short-season soybean accessions representing released cultivars and breeding lines from the active AAFC soybean breeding program at Ottawa Research and Development Centre over the last 30 years.

Approximately five seeds were randomly selected from each accession and planted in the greenhouse at the Saskatoon Research and Development Centre. Leaf tissue was collected at the 2–4 leaf stage separately for each plant, freeze-dried for 1–2 days in a Labconco (Kansas City, MO, USA) Freeze Dryer System, and stored at −20 °C. DNA was extracted from 12–15 mg of freeze-dried leaf tissue from one randomly selected plant per accession using the Qiagen BioSprint 96 DNA Plant Kit (Qiagen Inc., Toronto, ON, Canada), following the product handbook, with the exception of DNA elution in 100 µL rather than 200 µL of water. DNA quality was assessed using a 260/280-nm ratio from the Thermo Scientific Nanodrop 8000, and DNA was quantified using the Invitrogen Quant-iT^TM^ PicoGreen^®^ dsDNA Assay Kit (Life Technologies, Burlington, ON, Canada) and adjusted to 20 ng/µL with water.

### 2.2. Genotyping-by-Sequencing

We screened genome-wide genetic variability of soybean germplasm using the genetic diversity-focused GBS (gd-GBS) protocol described by Peterson et al. [20]. This protocol modified the original GBS method [21] in combination with the ddRAD procedure [22] to employ long-read Illumina sequencing and indexing for sample deconvolution. Six sequencing libraries, each having 95 samples and one technical replicate (i.e., one sample was present in all six libraries), were prepared following the methods described in Peterson et al. [20] with the following modifications. First, four new adapters with balanced (A/C, G/T) barcodes (Table S1) were applied evenly across the samples to increase sample diversity and avoid phasing read errors on the Illumina HiSeq. Second, ligated fragments were cleaned using only 1× Agencourt AMPure XP Beads (Beckman Coulter, Mississauga, ON) rather than 1.5×, and eluted in 40 µL rather than 30 µL reagent grade water. After preparation, libraries were quantified using a ddPCR ^TM^ Library Quantification Kit for Illumina TruSeq (Bio-Rad Laboratories, Inc., Hercules, CA, USA) and diluted to 6 pM. Libraries were sequenced by NRC (National Research Council, Saskatoon, SK, Canada) on a HiSeq 2500 using Rapid Mode, 1 × 250 bp (dual indexed). The sequencing generated 1152 sequence read files in FASTQ format and these raw sequences were deposited into the SRA database of the National Centre for Biotechnology Information (NCBI) under BioProject ID of PRJNA705793.

### 2.3. Bioinformatics Analysis

All FASTQ files were first assessed with FastQC [23] to determine if there was any 3′ adapter read-through; if so, such files were trimmed with Trimmomatic version 0.32 [24] using the following trim settings: ILLUMINACLIP:TruSeq3-PE-2.fa; SLIDINGWINDOW:10:24; and MINLEN:100 variables to remove any adapter sequence bases, trim bases where the average Phred score over a 10 base window was less than 24 and remove any sequences shorter than 100 bases, respectively. The statistics of raw and filtered sequence reads were generated using an in-house Perl script.

SNP calling was made for each sample from the trimmed FASTQ files against the soybean genome assembly of Wm82.a2.v1 [25,26]. The reference FASTA chromosome and scaffold names were modified with a custom Perl script to reflect the formatting required for our custom pipeline. Specifically, clean sequences of each sample were aligned to the reference using Bowtie2 version 2.2.6 [27] with the output in SAM format. Variant calling was done using SAMTOOLS version 0.1.18 [28] as follows: converted to BAM format with VIEW; sorted with SORT; variant identification with MPILEUP and converted to VCF format with BCFTOOLS VIEW. VCF file processing and final data output were generated using a collection of in-house Perl and Bash shell scripts referred to as the “referenceGeno” pipeline, which is available upon request to the corresponding author. These scripts helped to (1) filter the resulting SAMTOOLS VCF files to generate a list of potential SNPs in haplotype and genotype with 15%, 30%, and 50% missing data across all 576 accessions and (2) format them in different tab delimited tabular forms for different sequence analyses such as STRUCTURE and MEGA for further genetic analyses.

### 2.4. Genetic Diversity Analysis

SNP genotype data with up to 50% missing values were firstly cleaned with six control samples (one original sample plus its replicates in six HiSeq runs); only those markers having the same marker values for six control samples were kept. There was further removal of the markers with singletons, with the same genotype across the assayed samples and with more than three genotypes per sample. Minor allele frequency, the extent of missing SNPs, SNP distribution with respect to chromosome, country of origin and maturity were generated in a Microsoft Excel^®^ file.

The genetic structure of the 571 samples was analyzed based on the SNP data of 19,898 markers (or M50 dataset) using a model-based Bayesian method available in the program STRUCTURE version 2.2.3 [29]. The STRUCTURE program was run 30 times with 30-core parallel computing in a Linux server for each subgroup (K) value, ranging from 1 to 10, using the admixture model with 10,000 replicates for burn-in and 10,000 replicates during analysis. The final sample grouping was determined based on (1) the likelihood plot of these models, (2) the change in the second derivative (∆K) of the relationship between K and the log-likelihood [30], and (3) the stability of grouping patterns across 30 runs. For a given K with 30 runs, the run with the highest likelihood value was selected to assign the posterior membership coefficients to each sample. The posterior membership coefficients were displayed in a bar plot. The size and composition of each optimal cluster were analyzed with respect to sample origin and maturity.

The inferred genetic structure was further compared for consistency with the genetic relationships of individual samples obtained from two commonly applied approaches. A principal coordinate analysis (PCoA) of all 571 samples was performed using the R routine, AveDissR, for assessing genetic distinctness and redundancy [31,32] and plots of the first three resulting principal components were generated to assess the sample associations. A neighbor-joining (NJ) analysis of all 571 samples was also conducted using PAUP* [33] based on the SNP data of 19,898 markers and a radiation tree was displayed using MEGA 5.05 [34]. The resulting PCoA plots and NJ trees were individually labeled with respect to sample origin and maturity group.

An analysis of molecular variance (AMOVA) was performed with Arlequin version 3.01 [35] on 19,898 markers to quantify the genetic variation present among 24 groups of sample origin and among five maturity groups (Table 1). The corresponding pairwise genetic distances among the origin and maturity groups were also generated. Additional AMOVA was also made to quantify the genetic variation among genetic groups identified from the STRUCTURE and PCoA analyses.

### 2.5. Identifying Distinct and Redundant Germplasm

We applied the same approach as proposed by Fu [36] that was equivalent to the simple matching coefficient of Sokal and Michener [37] to calculate the pairwise genotypic dissimilarity and to generate the average pairwise dissimilarity (APD) per sample for the assessment of germplasm distinctness and redundancy. The higher the APD value, the more genetically distinct the sample is in the collection. The lower the APD value, the more genetically redundant the sample is in the collection. Ranking the APD values of all the assayed samples provides a means of identifying the most distinct and most redundant samples [36]. Specifically, we applied AveDissR to generate the APD value per sample. To verify the redundant and distinct samples identified, the PCoA plot was also made with the labels of the identified groups to determine their genetic associations with the whole assayed samples.

Extra effort was also made to generate a pairwise dissimilarity matrix for all 571 samples based on 19,898 markers using AveDissR with modification to output the matrix file. This analysis helped identify the sample pairs with the lowest pairwise dissimilarity values for the assessment of genetically duplicated samples.

## 3. Results

### 3.1. SNP Discovery

**Figure 1:**
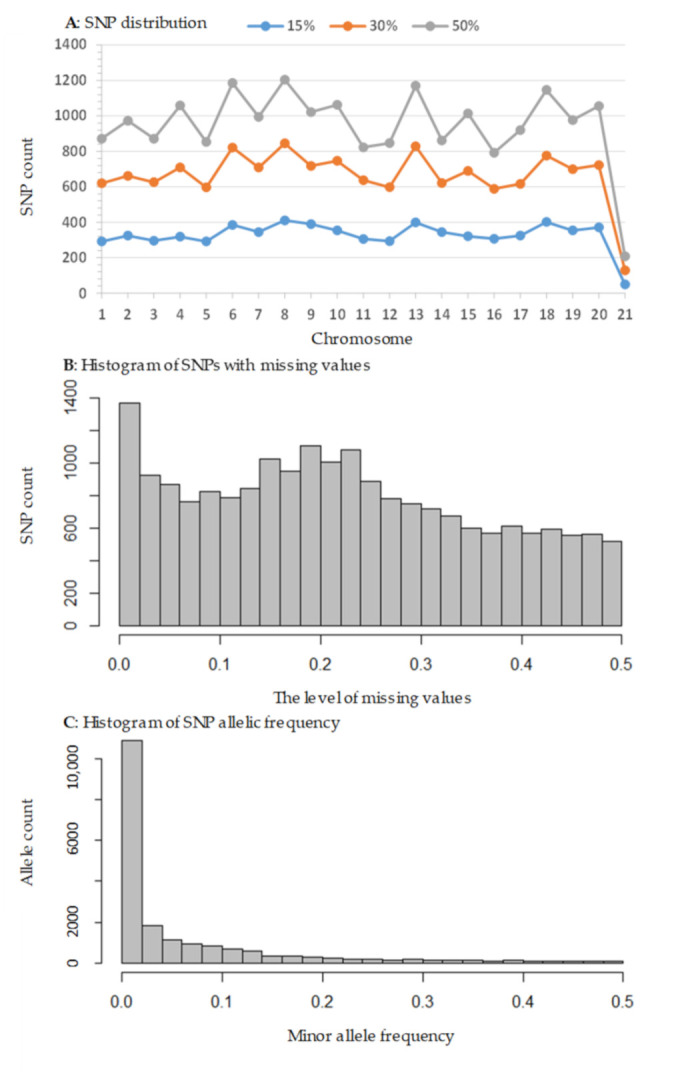
The frequency distribution of SNPs identified in this study with respect to chromosome (**A**), the level of missing values (**B**) and minor allele frequency (**C**). Panel (**A**) shows the SNP counts over all 20 chromosomes for SNPs with three levels of missing values (15%, 30% and 50%) across the 571 samples. Note that chromosome 21 represents all 69 scaffolds. Panel (**B**) displays the SNP counts with respect to missing value levels ranging from 0 to 50%. Panel (**C**) shows the minor allele frequency distribution in the dataset of SNPs with a 50% missing value level.

The HiSeq run of 576 soybean samples (Table S2) yielded approximately 548.4 million raw forward sequence reads from six libraries (Table S3). The number of raw forward sequence reads per sample ranged from 269,633 to 1,618,902 with an average of 952,074 and the filtered read counts varied from 255,484 to 1,521,313 with an average of 883,226 (Table S3). The SNP call detected 43,891 SNPs across the 576 samples on 20 chromosomes (43,161 SNPs) and 69 scaffolds (730 SNPs). As expected for the genotyping-by-sequencing technique, there were a large number of SNPs (or 19,318) having missing values for more than 50% assayed samples. Further removal of the same SNP genotype (2979 SNPs) or more than three genotypes (398 SNPs) across the assayed samples and inconsistent genotypes across the six repeat samples (1298 SNPs) generated three datasets of 19,898 SNPs with missing levels of 50% or less (M50 for short); 13,948 SNPs with 30% or less (M30); and 6861 SNPs with 15% or less (M15). The SNP distributions across the 20 chromosomes and 69 scaffolds for these three SNP datasets are shown in Figure 1A. The patterns of variation in SNP count per chromosome were similar for three missing value levels. On average, each chromosome had 985 SNPs at the M50 level, 691 SNPs at the M30 level, and 341 SNPs at the M15 level. Specific SNP distribution with respect to the level of missing value is illustrated in Figure 1B, and SNP counts generally decreased toward the increased level of missing value (up to 50%). Further characterization of the SNPs in the M50 dataset revealed that a majority of the SNPs had minor allelic frequency of 0.1 or lower (Figure 1C).

### 3.2. Patterns of Genetic Variability

**Figure 2:**
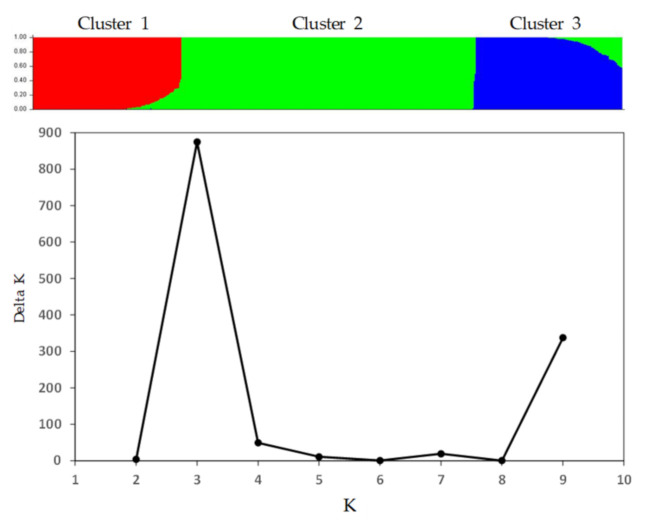
Three genetic clusters of 571 soybean samples inferred by STRUCTURE based on 19,898 SNP markers. The upper panel displays the sorted mixture coefficients of 571 samples with K = 3. The lower panel shows the support for three optimal clusters based on Delta K estimates.

The Bayesian inference of genetic structure by STRUCTURE without consideration of accession information revealed three optimal clusters of the 571 soybean samples with a strong delta K support (Figure 2). Clusters 1, 2, and 3 consisted of 144, 285, and 142 samples, respectively, and each cluster had mixed memberships originating from different countries (see Table S2 for the detailed memberships).

**Figure 3:**
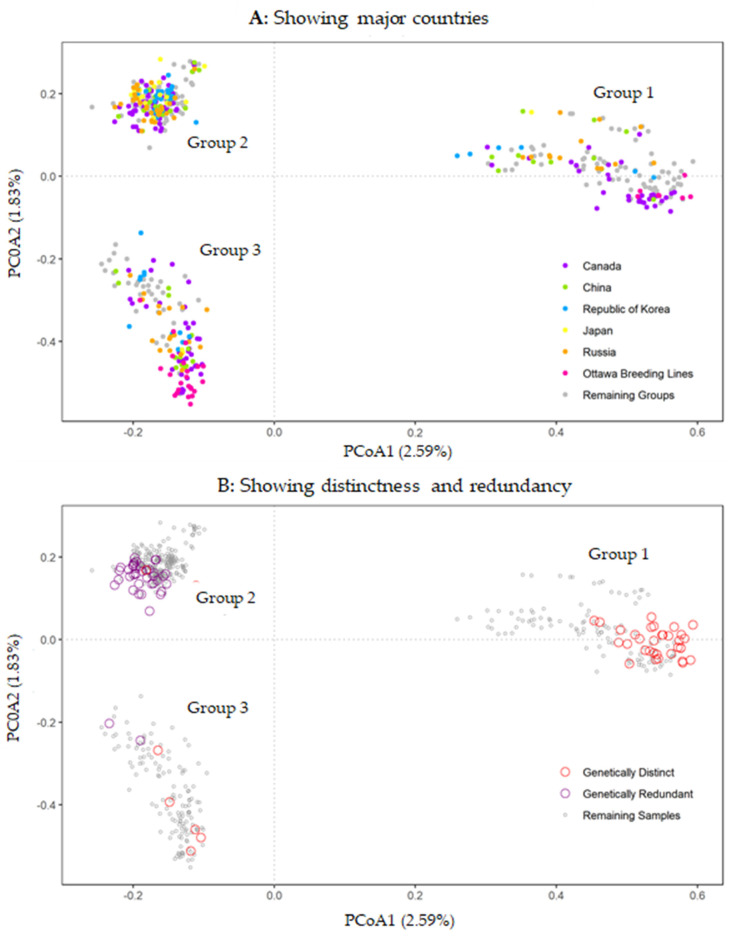
PCoA plots of 571 soybean accessions based on 19,898 SNP genotype data with a missing value level of up to 50%. Panel (**A**) displays the accessions originated from Canada, China, the Republic of Korea, Japan, and Russia, as highlighted in different colours, while the remaining groups are shown in grey. Three groups are also labelled. Panel (**B**) shows 40 genetically distinct samples in open red circles and 40 genetically redundant samples in open purple circles, while the remaining samples are shown in open grey circles.

The PCoA plot also revealed three distinct genetic groups of the 571 soybean samples (Figure 3). The membership of each group was the same as those inferred from STRUCTURE analysis, with one exception (see Table S2 for the sample membership). One sample in PCoA Group 3 had a membership coefficient of 0.504 for STRUCTURE Cluster 2 (vs. 0.496 for Cluster 3). Also, it was clear that the samples from Canada, China, the Republic of Korea, Japan and Russia were widely spread into three groups (Figure 3, Table 1 and Table S2). The 30 Ottawa breeding lines were located in Group 1 and Group 3 only. The first and second PCoA components explained 2.59% and 1.83% variances, respectively.

**Figure 4:**
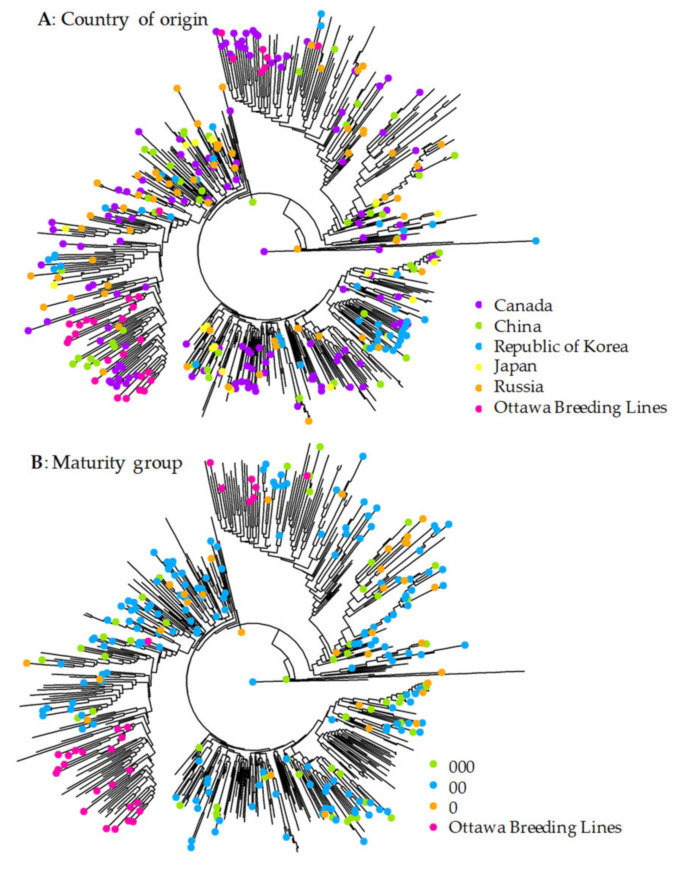
The neighbor-joining tree of 571 soybean accessions of different origins based on 19,898 SNP genotype data with a missing value level of up to 50%. Panels (**A**,**B**) are the same, but have different sample labels. Panel (**A**) shows the accessions originated from Canada, China, the Republic of Korea, Japan, and Russia. Panel (**B**) highlights three maturity groups and Ottawa breeding lines.

The NJ clustering revealed the same three distinct groups of all 571 soybean samples (Figure 4) as those inferred from STRUCTURE (Figure 2) and PCoA (Figure 3). However, the NJ tree revealed more sub-groups in each group and the mixture of sample origins in each group. There were no groups specific to samples originating from a specific or single country only (Figure 4A). Interestingly, all the samples of known maturity groups were also widely spread into three NJ groups, although the 30 Ottawa breeding lines (largely belonging to these three maturity groups) were located only in two NJ groups (Figure 4B), the same as in the PCoA plot (Figure 3). The NJ tree also helped to identify six genetically unique samples (CN32352, CN107502, CN45107, CN107642, CN36139, and CN107548). Quantifying the genetic variations of all 571 soybean samples representing 24 groups or countries of origin through AMOVA revealed 7.45% variance residing among these 24 groups and 92.55% variance present within groups (Table 2). The pairwise genetic distances among 24 groups ranged from 0 to 0.403 with a mean of 0.107, but only 109 out of 276 group pairs showed statistically significant distances (Table S4). This result indicates that the country of sample origin may not always be informative to identify genetically distinct samples. The AMOVA analysis also revealed that 5.55% variance was found to reside among five maturity groups (including Ottawa breeding lines and an unknown origin group) and 94.45% within the maturity groups. The pairwise group distances were not statistically significant among three maturity groups, but these three groups had significant pairwise group distances with the Ottawa breeding lines (e.g., 0.235 between group 000 and Ottawa breeding lines). Further AMOVA analysis of three PCoA groups revealed 34.52% variance resided among the inferred three groups and 65.48% was present within the groups. The largest significant pairwise distance was 0.413 between Group 1 and Group 3, followed by those between Groups 1 and 2 (0.363) and between Groups 2 and 3 (0.282).

### 3.3. Genetic Distinctness and Redundancy

The APD estimates for the 571 assayed samples ranged from 0.0986 to 0.2522 with a mean of 0.1284 and standard deviation of 0.018 (Table S2). The frequency distribution of these APD values is given in Figure S1A, and there were 2, 19 and 106 samples with APD estimates greater than 3, 2 and 1 standard deviation, respectively, while there were only 72 samples with APD estimates lower than one standard deviation. The regression analyses of the APD estimates over the three levels of SNP missing values (50%, 30% and 15%) revealed that the correlations among those APD estimates were high, ranging from 0.813 to 1.027 with R2 greater than 0.902 (Figure S1).

As the APD value of a particular sample measures the overall genetic difference of the sample against the remaining samples of the collection, we selected 40 more genetically distinct samples with APD estimates greater than 0.1565 and 40 more genetically redundant samples with APD estimates lower than 0.1076 for further germplasm management (Table 3). Note that the 40 samples with the most genetic distinctness included the first three distinct samples identified from the NJ tree (Figure 4). The genetic relationships of the selected 80 samples with the remaining samples are also displayed in Figure 3B. The selected genetically distinct samples spread over into three groups, but were mainly located in Group 1, while the selected genetically redundant samples mainly harbored in Group 2, with two samples placed in Group 3. The frequency distributions of these 80 selected samples with respect to origin and maturity are shown in Table 1.

We also identified 16 sample pairs with the lowest pairwise dissimilarity values that were roughly equivalent to 37 or fewer (out of 19,898) SNP markers showing genetic differences between two samples (Table 4). Such extremely low pairwise dissimilarity values clearly indicate the presence of genetic redundancy. The 16 (out of 162,735) sample pairs had only 22 samples, of which 13 were considered as genetically duplicated samples (Table 4). More specifically, each of the 13 samples was at least 99.8% identical, based on 19,898 SNP markers, to at least one other sample.

## 4. Discussion

The germplasm characterization presented here revealed some interesting patterns of genetic variation in the soybean collection held at Plant Gene Resources of Canada. First, there were three distinct genetic groups present in the assayed samples, and each group was a mixture of the samples originating from different countries and with different known maturity groups (Table 1 and Figure 3). Second, the samples originating from Canada were clustered in all three genetic groups, but the 30 Ottawa breeding lines were clustered only in two groups (Table 1 and Figure 4). Third, the 40 samples with the most genetic distinctness represented three genetic groups with diverse sample origin and known maturity (Table 3). In contrast, the 40 samples with the highest genetic redundancy consisted of different sample origins and maturity groups, but were largely from one genetic group. Fourth, the extent of genetic duplication was relatively low in the collection and only 13 samples were identified as genetically duplicated samples (Table 4). These findings are useful for soybean germplasm management and utilization.

Three different diversity analyses (Figure 2, Figure 3 and Figure 4) revealed the presence of three distinct genetic groups of soybean germplasm in the PGRC soybean collection, but each of these groups was well mixed with diverse sample origins and not unique to germplasm from China, Korea, Japan or Russia. This was unexpected, as soybean is thought to have been domesticated in China around the eleventh century BCE and then disseminated to surrounding countries around the first century CE [38]. Also, it is not consistent with those distinct clusters reported by Song et al. [10] of soybean wild and landrace genotypes from different countries, in which genetic clusters were well aligned with the sample origins (see Figure 1 of Song et al. [10]). One possible explanation is that the inferred genetic groups reflects the unique gene pool generated by the Canadian soybean breeding programs over the last 80 years [39,40], as the PGRC soybean collection was largely acquired from those Canadian breeding programs aimed for improving short-season soybean. Also, the known maturity groups were well presented in the inferred genetic groups (Table 3). However, the diversity analyses of the Canadian soybean cultivars released over the last 80 years did not reveal marked genetic groups [36,41]. Clearly, more research is needed to understand the origin of these distinct genetic groups.

We applied pairwise dissimilarity to identify genetic duplicates and average pairwise dissimilarity to identify the samples with the most genetic redundancy. Overall, the extent of genetic duplication in the PGRC soybean collection was relatively low (Table 3 and Table 4). This finding, however, is inconsistent with those reported for the USDA soybean collection [10], but it is not surprising either, given the collection history and acquisition sources. However, such duplication identification was not without limitations [36]. It depends on the number of samples assayed and the SNP markers used. More samples would enhance the power of identification. Fewer genome-wide SNP markers with missing values could also affect the reliability of estimating average pairwise dissimilarity (see Figure S1). Also, the most genetically redundant samples were relative to the whole set of samples assayed, and there is no solid threshold to group samples with and without genetic redundancy. Moreover, we did not examine within-accession variation, as such variation was expected to be low [42] for soybean with an outcrossing rate of 1–2% [43,44]. In spite of this, we cannot rule out the effects, if any, of the within-accession variation on the estimation of pairwise dissimilarity and duplication identification. Thus, these identifications may not necessarily identify the true duplicated accessions per se [36], but suggest the potential duplicate candidates for germplasm management.

Our characterization also generated a unique set of genomic resources for genetic analyses of soybean germplasm. The acquired SNP genotype data had an adequate genome sampling across the 20 chromosomes (Figure 1A) and can be applied through a genome-wide association analysis to identify genetic regions associated with various traits of breeding targets such as agronomic traits, early maturity, and quality traits, if those assayed accessions are phenotypically evaluated. These efforts will facilitate the search for genetic variants of breeding importance from the conserved germplasm. Also, genotyping-by-sequencing is known to generate SNP genotypes with large amounts of missing data [45], as variation could occur in the restriction site during library preparation, PCR bias during library amplification, and/or flow-cell sequencing bias due to increased multiplexing. However, our preliminary analysis seemed to suggest that the SNP genotypes with up to 50% level of missing values across the samples were still informative, at least for estimating genetic diversity (Figure S1). Further diversity analyses based on the M30 and M15 datasets revealed the same three distinct genetic groups, although the group distinctness was slightly reduced (or more dispersed) for the M15 dataset (results not shown). The patterns of variation with respect to sample origin and maturity group remained essentially the same as for the M50 dataset.

The revealed patterns of genetic variation have implications for managing soybean germplasm. The genetic variation was relatively low with respect to sample origin and maturity group, but considerably larger among three distinct genetic groups (Table 2; Figure 3). Thus, some attention should be paid to manage these three distinct genetic groups. The genetic distinctness measured with average pairwise dissimilarity can be informative to the development of core subsets of soybean germplasm from the collection for germplasm utilization, as the identified samples can serve as the candidates for further consideration, including the field evaluation on the traits of breeding targets. This could be achieved following the same integrated approach for the development of the flax core collection [46]. Also, the revealed genetic distinctness can be useful for the selection of a set of distinct soybean germplasm for safety backup in other genebanks. As mentioned above, the genetic redundancy in the collection was relatively low, as the lowest average pairwise dissimilarity values had just one standard deviation apart from the mean, and those redundancies mainly resided in the genetic group 2. The identified genetic duplicates can be further verified in the field to determine their accession duplication.

The findings presented here also have some implications for germplasm utilization in soybean breeding. Breeders searching for germplasm with early maturity need to screen all the soybean accessions in the collection, as the inferred genetic groups were not unique to specific maturity groups, nor to specific sample origins. A field evaluation of maturity traits is needed. Identification and exploration of genes associated with early maturity traits can proceed with any genetic group, but would be more fruitful with the group in which the majority of the 30 Ottawa breeding lines resided.

## 5. Conclusions

The genotyping-by-sequencing generated abundant SNP markers across the 20 soybean chromosomes. The diversity analysis revealed three distinct genetic groups present in the soybean collection held in Plant Gene Resources of Canada. These groups were a mixture of the samples originating from different countries and samples of known maturity groups. The 30 Ottawa breeding lines were clustered with two of the three groups. The analysis also identified 40 samples with the most genetic distinctness and 40 samples with the highest genetic redundancy and showed that the extent of genetic duplication in the collection was relatively low. These findings are useful for soybean germplasm management and utilization.

## Figures and Tables

**Table 1:** Frequency distribution of 571 soybean samples with respect to country of origin, maturity group, PCoA-group and average pairwise dissimilarity (APD).

		Total		PCoA-Group		Average Pairwise Dissimilarity	
Group	Label	Count	1	2	3	Highest 40	Lowest 40
*Country of origin*							
Canada	1	130	32	60	38	3	15
Russian Federation	2	61	13	30	18	2	6
China	3	46	11	22	13	0	3
Sweden	4	46	9	29	8	3	2
Korea, Republic Of	5	44	6	27	11	3	3
Germany	6	38	17	16	5	8	2
France	7	26	10	14	2	2	0
Japan	8	19	1	16	2	0	0
Hungary	9	16	6	10	0	1	0
Poland	10	23	3	14	6	1	4
Unknown	11	22	5	12	5	2	1
Romania	12	11	5	5	1	2	1
Netherlands	13	14	3	10	1	0	0
Belgium	14	9	3	2	4	1	0
United States	15	5	0	4	1	1	1
Switzerland	16	4	1	3	0	1	1
Ukraine	17	4	0	3	1	0	0
United Kingdom	18	4	1	1	2	0	0
Yugoslavia	19	4	1	1	2	0	0
Austria	20	3	2	1	0	2	1
Bulgaria	21	3	2	1	0	1	0
Italy	22	2	2	0	0	1	0
Czech Republic	23	1	0	1	0	0	0
Serbia	23	1	1	0	0	1	0
Lithuania	23	1	1	0	0	0	0
Moldova	23	1	0	1	0	0	0
Norway	23	1	0	1	0	0	0
Portugal	23	1	1	0	0	0	0
Slovakia	23	1	1	0	0	0	0
Ottawa Breeding Lines	24	30	7	0	23	5	0
Total	24	571	144	284	143	40	40
*Maturity group*							
000	1	51	13	25	13	4	2
00	2	159	46	77	36	7	14
0	3	33	14	15	4	3	4
Ottawa Breeding Lines	4	30	7	0	23	5	0
Unknown	5	298	64	167	67	21	20

**Table 2:** Results for analysis of molecular variance based on 19,898 SNP markers in 571 soybean accessions representing 29 countries (including Ottawa soybean breeding lines and one group of unknown origin), 5 maturity groups and three groups inferred from PCoA.

Model/Source	*df*	Sum of Squares	Variance Component	Percent of Variation	*p*-Value
*Country and region*					
Among countries	23	75539.69	57.42	7.45	<0.00001
Within countries	1118	797242.03	713.1	92.55	
*Maturity group*					
Among groups	4	34431.26	43.34	5.55	<0.00001
Within groups	1137	838350.46	737.34	94.45	
*Three groups inferred by PCoA*					
Among groups	2	217898.37	303.04	34.52	<0.00001
Within groups	1139	654883.36	574.96	65.48	

**Table 3:** List of 40 genetically distinct and 40 genetically redundant soybean accessions identified based on average pairwise dissimilarity (APD) values of 571 assayed accessions.

Genetic Distinctness		Origin	CG	MG	PG	APD	Genetic Redundancy		Origin	CG	MG	PG	APD
Sample	Description						Sample	Description					
CN32352	No. 854	KOR	5	5	2	0.2522	CN52636	CH20731	UNK	11	5	2	0.0986
CN107502	B 10	DEU	6	3	3	0.1960	CN115251		SWE	4	5	2	0.0997
CN107476	J-40	HUN	9	2	1	0.1786	CN33265	OX719	CAN	1	5	2	0.0998
CN107514	N 19	FRA	7	2	1	0.1775	CN107577	Shika No. 4	CAN	1	2	2	0.0998
CN107495	Strain No. 196	DEU	6	2	1	0.1767	CN107575	Honshu No. 4	CHN	3	3	2	0.0999
CN107498	Strain No. 142	DEU	6	1	1	0.1750	CN115253		USA	15	5	2	0.1002
CN29751	Jin Nung No. 5	ROM	12	5	1	0.1716	CN36334	Rekord Severnyj	RUS	2	5	2	0.1008
CN35339	KAS351-4	KOR	5	5	1	0.1710	CN115258		POL	10	5	2	0.1017
CN45107	WIR 5683	USA	15	5	2	0.1706	CN107579	(F59-244)	CAN	1	2	2	0.1024
CN39084	X698-5-1	CAN	1	5	3	0.1699	CN33911	Hei Nung No. 26	KOR	5	5	2	0.1025
CN107522	738-3	SWE	4	2	1	0.1694	CN107571	Mandurska 2	CAN	1	3	2	0.1032
CN52877	No. 536	UNK	11	5	1	0.1693	CN33253	Morsoy	CHE	16	5	2	0.1034
CN107496	Strain No. 184	DEU	6	2	1	0.1685	CN107555	Amurszkaja 41	RUS	2	3	2	0.1035
CN32766	No. 601	DEU	6	5	1	0.1680	CN107566	Record North	CAN	1	2	2	0.1040
CN107497	Strain No. 134	DEU	6	1	1	0.1672	CN107581	(Iregy soja)	CAN	1	2	2	0.1042
CN39075	X691-3-1	UNK	11	5	1	0.1664	CN35918	Saliut 216	RUS	2	5	2	0.1045
CN32662	No. 547	AUT	20	5	1	0.1660	CN107588	PI 358321c	CAN	1	2	2	0.1046
CN107839	Szaljut	CHE	16	3	1	0.1658	CN107855	754-5	AUT	20	2	2	0.1047
CN32416	No. 1038	AUT	20	5	1	0.1655	CN107813	698-3-5	SWE	4	2	2	0.1047
CN35364	KAS629-1	KOR	5	5	1	0.1638	CN33248	Harosoy 63	CAN	1	5	2	0.1047
CN107662	Ainushyi 262	RUS	2	5	3	0.1619	CN107538	766-2	POL	10	2	3	0.1049
CN30642	ISZ 10	DEU	6	5	1	0.1612	CN107425	Accord	CAN	1	5	2	0.1052
CN32320	No. 940	ITA	22	5	1	0.1608	CN33255	Vansoy	CAN	1	5	2	0.1054
QGC10N	QGC10N	OBL	24	4	3	0.1605	CN107572	Urozsajnaja	CAN	1	3	2	0.1054
CN107518	Halton	FRA	7	2	1	0.1603	CN107614	Hercumft	DEU	6	1	2	0.1054
Canatto	Canatto	OBL	24	4	1	0.1600	CN45090	150	ROM	12	5	2	0.1056
CN35313	KAS134-2	SRB	23	5	1	0.1598	CN36218	Hei 3-18	CHN	3	5	2	0.1061
CN39077	X691-12-1	CAN	1	5	3	0.1597	CN107585	PI 358320	CAN	1	2	2	0.1062
AACUmami	AAC Umami	OBL	24	4	1	0.1591	CN107580	Iregy soja	CAN	1	2	2	0.1062
CN107523	738-4	SWE	4	1	1	0.1590	CN107421	RCAT Bobcat	CAN	1	5	2	0.1063
CN107838	Pannonia 10	BEL	14	3	1	0.1588	CN29792	Feng Shou No. 12	RUS	2	5	2	0.1064
CN107527	748-7	SWE	4	1	1	0.1588	CN33273	Beechwood	KOR	5	5	2	0.1065
Nattosan	Nattosan	OBL	24	4	1	0.1585	CN39173	X879-17-B	POL	10	5	2	0.1065
CN32394	PGR 3866	POL	10	5	1	0.1585	CN107422	Mario	CAN	1	5	2	0.1067
CN35327	KAS202-1	RUS	2	5	1	0.1584	CN107546	38777	RUS	2	2	2	0.1067
CN107360	Sara	CAN	1	5	1	0.1583	CN107624	Zarja	KOR	5	2	3	0.1069
CN29789	Hei Nung No. 18	BGR	21	5	1	0.1578	CN35314	KAS134-5	CHN	3	5	2	0.1073
AACSpringfield	AAC Springfield	OBL	24	4	1	0.1573	CN107882	Starachramiskaya	RUS	2	2	2	0.1073
CN30316	Early Harvest No. 1	ROM	12	5	1	0.1568	CN107644	Zolta z Zolna	POL	10	2	2	0.1075
CN107494	Strain No. 28	DEU	6	2	1	0.1565	CN107550	Soja-C.-St. 12/58	DEU	6	1	2	0.1075

Note that origin of country is given in the ISO 639-2 code; UNK is for unknown and OBL is for the AAFC Ottawa breeding line. CG stands for country or group (see Table 1). MG is for five maturity groups and PG for three PCoA-based groups (see Table S2 or Figure 3).

**Table 4:** List of 16 sample pairs having extremely low pairwise dissimilarity values (or equivalent to 37 or fewer different loci out of 19,898 SNP markers) and the number of genetically duplicated (GD) samples.

Sample Pair		Pairwise Dissimilarity	Equivalent to Different Loci	Number of Samples	Number of GD Samples
Sample 1	Sample 2				
*A group of five sample pairs*				4	3
CN32257	CN31690	0.00130	26		
CN32257	CN31719	0.00130	26		
CN32257	CN32634	0.00152	30		
CN31690	CN32634	0.00097	19		
CN31690	CN31719	0.00149	30		
*A group of four sample pairs*				4	3
CN107815	CN107819	0.00119	24		
CN107815	CN31984	0.00187	37		
CN107815	CN107461	0.00133	26		
CN107819	CN31984	0.00157	31		
*Individual sample pair*					
CN35309	CN35329	0.00096	19	2	1
CN32829	CN32451	0.00143	29	2	1
CN32053	CN32631	0.00112	22	2	1
CN107826	CN31692	0.00169	34	2	1
CN107580	CN107581	0.00085	17	2	1
CN107562	CN107631	0.00166	33	2	1
CN107558	CN107855	0.00152	30	2	1
Total				22	13

Note that any three of the four samples in each group of sample pairs and any one sample in individual sample pairs could be considered as genetically duplicated samples to at least one other sample.

## Data Availability

The original DNA sequence data generated for this study were deposited into the SRA database of the National Centre for Biotechnology Information under BioProject ID of PRJNA705793.

## Supplementary Materials

The following are available online at https://www.mdpi.com/article/10.3390/plants10081611/s1, Figure S1: Average pairwise dissimilarity (APD) for three levels of SNP missing value (50%, 30% and 15%) (A: APD50, B: APD30 and C: APD15) and their correlations (D: APD30 vs APD50, E: APD15 vs APD50, F: APD15 vs APD30), Table S1: List of four new adaptors with sequence information used in the genotyping-by-sequencing procedure, Table S2: List of 571 soybean accessions with the country of origin, country group (CG), maturity group (MG), average pairwise dissimilarity (APD), PCoA-group (PG) and STRUCTURE-cluster (SC), Table S3: Sequence summary for 571 assayed soybean samples, including NCBI accession information, Table S4: The pairwise group genetic distances obtained from AMOVA analysis of 24 countries or groups (in the lower diagonal).
