# Supplementary material for: Patterns of Genetic Variation in a Soybean Germplasm Collection as Characterized with Genotyping-by-Sequencing
Source: Plants (Basel). 2021 Aug 5;10(8):1611. doi: 10.3390/plants10081611 (PMC8399144; doi:10.3390/plants10081611)
Supplement: Supplementary file 1 [file plants-10-01611-s001.zip › plants-1317515 - supplementary.pdf]

plants

Supplemental materials

Patterns of Genetic Variation in a Soybean Germplasm Collection as Characterized with Genotyping-by-Sequencing

Yong-Bi Fu [1,*], Elroy R. Cober [2], Malcolm J. Morrison [2], Frédéric Marsolais [3], Gregory W. Peterson [1] and Carolee Horbach [1]

[1] Plant Gene Resources of Canada, Saskatoon Research and Development Centre, Agriculture and Agri-Food Canada, 107 Science Place, Saskatoon, SK S7N 0X2, Canada; gregory.peterson@agr.gc.ca (G.W.P.); carolee.horbach@agr.gc.ca (C.H.)
[2] Ottawa Research and Development Centre, Agriculture and Agri-Food Canada, Ottawa, ON K1A 0C6, Canada; elroy.cober@agr.gc.ca (E.R.C.); malcolm.morrison@agr.gc.ca (M.J.M.)
[3] Genomics and Biotechnology, London Research and Development Centre, Agriculture and Agri-Food Canada, London, ON N5V 4T3, Canada; frederic.marsolais@agr.gc.ca
* Correspondence: yong-bi.fu@agr.gc.ca

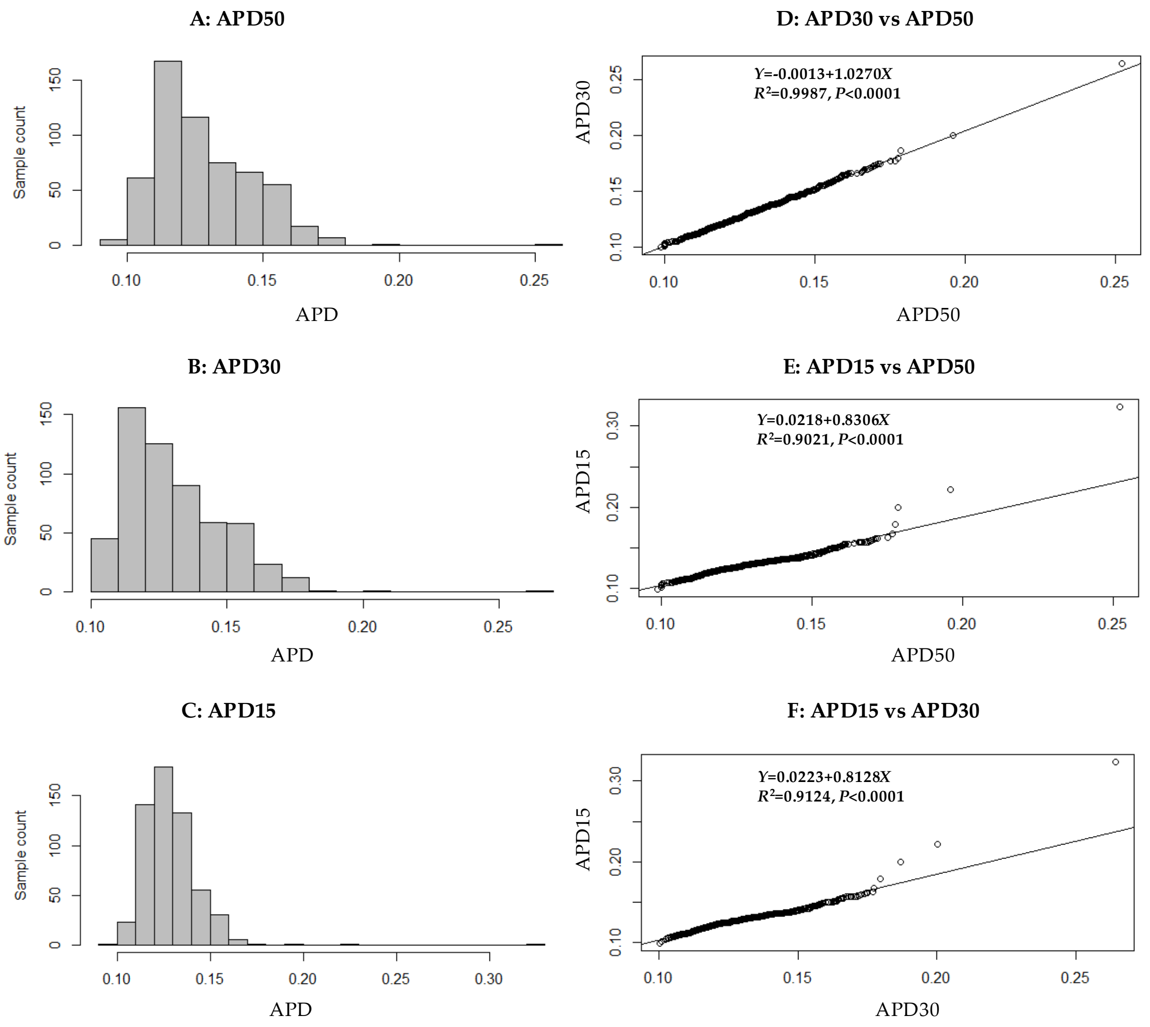

Figure S1. Average pairwise dissimilarity (APD) for three levels of SNP missing value (50%, 30% and 15%) (**A**: APD50, **B**: APD30 and **C**: APD15) and their correlations (**D**: APD30 vs APD50, **E**: APD15 vs APD50, **F**: APD15 vs APD30).

Table S1. List of four new adaptors with sequence information used in the genotyping-by-sequencing procedure.

**Adaptor**	**Sequence (5' to 3')**
*Pst*I_CGACG_top	ACA CTC TTT CCC TAC ACG ACG CTC TTC CGA TCT CGA CGT GCA
*Pst*I_CGACG_bottom	/5Phos/CGT CGA GAT CGG AAG AGC GTC GTG TAG GGA AAG AGT GTA GAT C
*Pst*I_GATGA_top	ACA CTC TTT CCC TAC ACG ACG CTC TTC CGA TCT GAT GAT GCA
*Pst*I_GATGA_bottom	/5Phos/TCA TCA GAT CGG AAG AGC GTC GTG TAG GGA AAG AGT GTA GAT C
*Pst*I_AATCG_top	ACA CTC TTT CCC TAC ACG ACG CTC TTC CGA TCT AAT CGT GCA
*Pst*I_AATCG_bottom	/5Phos/CGA TTA GAT CGG AAG AGC GTC GTG TAG GGA AAG AGT GTA GAT C
*Pst*I_GTATA_top	ACA CTC TTT CCC TAC ACG ACG CTC TTC CGA TCT GTA TAT GCA
*Pst*I_GTATA_bottom	/5Phos/TAT ACA GAT CGG AAG AGC GTC GTG TAG GGA AAG AGT GTA GAT C

Table S2. List of 571 soybean accessions with the country of origin, country group (CG), maturity group (MG), average pairwise dissimilarity (APD), PCoA-group (PG) and STRUCTURE-cluster (SC).

Key	Sample	Origin	CG	MG	APD	PG	SC	Key	Sample	Origin	CG	MG	APD	PG	SC	Key	Sample	Origin	CG	MG	APD	PG	SC
1	CN29752_S1	HUN	9	5	0.1254	2	2	97	CN32351_S1	JPN	8	5	0.1340	2	2	193	CN30633_S1	RUS	2	5	0.1206	2	2
2	CN29792_S2	RUS	2	5	0.1064	2	2	98	CN35348_S2	HUN	9	5	0.1274	2	2	194	CN30634_S2	CHN	3	5	0.1199	2	2
3	CN29793_S3	CHE	16	5	0.1077	2	2	99	CN35373_S3	ROM	12	5	0.1280	2	2	195	CN31690_S3	CHN	3	5	0.1215	2	2
4	CN29795_S4	DEU	6	5	0.1126	2	2	100	CN42821_S4	CHE	16	5	0.1185	2	2	196	CN31692_S4	CHN	3	5	0.1112	2	2
5	CN30389_S5	DEU	6	5	0.1205	2	2	101	CN107435_S5	CZE	23	5	0.1188	2	2	197	CN32053_S5	CHN	3	5	0.1087	2	2
6	CN30391_S6	NLD	13	5	0.1185	2	2	102	CN107437_S6	KOR	5	5	0.1153	2	2	198	CN32257_S6	RUS	2	5	0.1306	2	2
7	CN32356_S7	YUG	19	5	0.1138	2	2	103	CN107650_S7	KOR	5	5	0.1233	2	2	199	CN32442_S7	RUS	2	5	0.1120	2	2
8	CN32451_S8	GBR	18	5	0.1128	2	2	104	CN107804_S8	KOR	5	3	0.1272	2	2	200	CN32634_S8	HUN	9	5	0.1272	2	2
9	CN32547_S9	DEU	6	5	0.1346	2	2	105	CN107822_S9	CAN	1	3	0.1194	2	2	201	CN32639_S9	DEU	6	5	0.1116	2	2
10	CN32826_S10	DEU	6	5	0.1102	2	2	106	CN107879_S10	CAN	1	2	0.1266	2	2	202	CN32829_S10	UKR	17	5	0.1078	2	2
11	CN33248_S11	CAN	1	5	0.1047	2	2	107	CN107880_S11	RUS	2	2	0.1268	2	2	203	CN33253_S11	CHE	16	5	0.1034	2	2
12	CN33259_S12	CAN	1	5	0.1134	2	2	108	CN107881_S12	RUS	2	1	0.1256	2	2	204	CN33255_S12	CAN	1	5	0.1054	2	2
13	CN35309_S13	CAN	1	5	0.1215	2	2	109	CN107882_S13	RUS	2	2	0.1073	2	2	205	CN33265_S13	CAN	1	5	0.0998	2	2
14	CN35312_S14	CAN	1	5	0.1167	2	2	110	CN107883_S14	RUS	2	1	0.1081	2	2	206	CN33273_S14	KOR	5	5	0.1065	2	2
15	CN35314_S15	CHN	3	5	0.1073	2	2	111	CN107884_S15	CAN	1	2	0.1128	2	2	207	CN33911_S15	KOR	5	5	0.1025	2	2
16	CN35321_S16	KOR	5	5	0.1200	2	2	112	CN107885_S16	DEU	6	2	0.1127	2	2	208	CN35308_S16	KOR	5	5	0.1211	2	2
17	CN35329_S17	KOR	5	5	0.1196	2	2	113	CN115251_S17	SWE	4	5	0.0997	2	2	209	CN35318_S17	KOR	5	5	0.1164	2	2
18	CN35380_S18	KOR	5	5	0.1199	2	2	114	CN115252_S18	USA	15	5	0.1105	2	2	210	CN35372_S18	KOR	5	5	0.1206	2	2
19	CN35919_S19	KOR	5	5	0.1118	2	2	115	CN115253_S19	USA	15	5	0.1002	2	2	211	X0CN35382_S19	KOR	5	5	0.1134	2	2
20	CN36138_S20	UKR	17	5	0.1181	2	2	116	CN115254_S20	JPN	8	5	0.1168	2	2	212	CN35757_S20	RUS	2	5	0.1213	2	2
21	CN36143_S21	MDA	23	5	0.1125	2	2	117	CN115255_S21	POL	10	5	0.1131	2	2	213	CN35758_S21	CAN	1	5	0.1243	2	2
22	CN36334_S22	RUS	2	5	0.1008	2	2	118	CN115256_S22	DEU	6	5	0.1140	2	2	214	CN35916_S22	CAN	1	5	0.1351	2	2
23	CN52642_S23	RUS	2	5	0.1124	2	2	119	CN115257_S23	POL	10	5	0.1103	2	2	215	CN35918_S23	RUS	2	5	0.1045	2	2
24	CN107446_S24	UKR	17	5	0.1104	2	2	120	CN115258_S24	POL	10	5	0.1017	2	2	216	CN35920_S24	RUS	2	5	0.1234	2	2
25	CN107450_S25	RUS	2	5	0.1371	3	3	121	CN115259_S25	POL	10	5	0.1497	3	3	217	CN35921_S25	CAN	1	5	0.1238	2	2
26	CN107455_S26	CAN	1	5	0.1252	3	3	122	MapleArrow_S26	OBL	24	4	0.1352	3	3	218	CN36139_S26	CAN	1	5	0.1417	2	2
27	CN107477_S27	CAN	1	2	0.1406	3	3	123	MapleGlen_S27	OBL	24	4	0.1340	3	3	219	CN36141_S27	CAN	1	5	0.1096	2	2
28	CN107479_S28	RUS	2	2	0.1412	3	3	124	ACOrford_S28	OBL	24	4	0.1380	3	3	220	CN36335_S28	UNK	11	5	0.1188	2	2
29	CN107486_S29	JPN	8	2	0.1445	3	3	125	Accord_S29	OBL	24	4	0.1469	3	3	221	CN36337_S29	BEL	14	5	0.1195	2	2
30	CN107501_S30	RUS	2	2	0.1352	3	3	126	RD714_S30	OBL	24	4	0.1164	3	3	222	CN36340_S30	NLD	13	5	0.1138	2	2
31	CN107511_S31	DEU	6	2	0.1255	3	3	127	ACGlengarry_S31	OBL	24	4	0.1334	3	3	223	CN36342_S31	FRA	7	5	0.1151	2	2
32	CN107524_S32	CAN	1	1	0.1455	3	3	128	DH3604_S32	OBL	24	4	0.1513	3	3	224	CN39070_S32	FRA	7	5	0.1124	2	2
33	CN107529_S33	USA	15	2	0.1220	3	3	129	Toki_S33	OBL	24	4	0.1547	3	3	225	CN42928_S33	SWE	4	5	0.1133	2	2
34	CN107590_S34	UKR	17	3	0.1312	3	3	130	Chikala_S34	OBL	24	4	0.1312	3	3	226	CN45087_S34	SWE	4	5	0.1270	2	2
35	CN107598_S35	YUG	19	3	0.1547	3	3	131	QGC10N_S35	OBL	24	4	0.1605	3	3	227	CN45088_S35	RUS	2	5	0.1482	2	2
36	CN107630_S36	GBR	18	2	0.1379	3	3	132	QGC12N_S36	OBL	24	4	0.1552	3	3	228	CN45090_S36	ROM	12	5	0.1056	2	2
37	CN107652_S37	DEU	6	5	0.1307	3	3	133	Dares_S37	OBL	24	4	0.1269	3	3	229	CN45095_S37	JPN	8	5	0.1114	2	2
38	CN107654_S38	RUS	2	5	0.1381	3	3	134	Apalis_S38	OBL	24	4	0.1529	3	3	230	CN45107_S38	USA	15	5	0.1706	2	2
39	CN107655_S39	JPN	8	5	0.1384	3	3	135	Loriot_S39	OBL	24	4	0.1505	3	3	231	CN51375_S39	UNK	11	5	0.1218	2	2
40	CN107656_S40	RUS	2	5	0.1392	3	3	136	Jari_S40	OBL	24	4	0.1453	3	3	232	CN52633_S40	SWE	4	5	0.1109	2	2
41	CN107657_S41	RUS	2	5	0.1330	3	3	137	DH710_S41	OBL	24	4	0.1418	3	3	233	CN52634_S41	SWE	4	5	0.1136	2	2
42	CN107658_S42	RUS	2	5	0.1252	3	3	138	SG1010_S42	OBL	24	4	0.1409	3	3	234	CN52635_S42	UNK	11	5	0.1092	2	2
43	CN107659_S43	RUS	2	5	0.1270	3	3	139	Roselin_S43	OBL	24	4	0.1437	3	3	235	CN52639_S43	CHN	3	5	0.1134	2	2
44	CN107660_S44	RUS	2	5	0.1368	3	3	140	AACVireo_S44	OBL	24	4	0.1408	3	3	236	CN52640_S44	UNK	11	5	0.1131	2	2
45	CN107661_S45	RUS	2	5	0.1264	3	3	141	AACEdward_S45	OBL	24	4	0.1316	3	3	237	CN52643_S45	UNK	11	5	0.1086	2	2
46	CN107662_S46	RUS	2	5	0.1619	3	3	142	AACNaruto_S46	OBL	24	4	0.1226	3	3	238	CN52645_S46	UNK	11	5	0.1101	2	2
47	CN107663_S47	RUS	2	5	0.1384	3	3	143	Heron_S47	OBL	24	4	0.1415	3	3	239	CN52646_S47	UNK	11	5	0.1101	2	2
48	CN107664_S48	RUS	2	5	0.1193	3	3	144	ACColibri_S48	OBL	24	4	0.1379	3	3	240	CN52647_S48	RUS	2	5	0.1100	2	2
49	CN107666_S49	RUS	2	5	0.1472	1	1	145	Nattosan_S49	OBL	24	4	0.1585	1	1	241	CN52648_S49	RUS	2	5	0.1229	1	1
50	CN107667_S50	RUS	2	5	0.1472	1	1	146	Canatto_S50	OBL	24	4	0.1600	1	1	242	CN52649_S50	RUS	2	5	0.1273	1	1
51	CN107668_S51	RUS	2	5	0.1249	1	1	147	AACUmami_S51	OBL	24	4	0.1591	1	1	243	CN52650_S51	RUS	2	5	0.1249	1	1
52	CN107800_S52	RUS	2	5	0.1455	1	1	148	AACSpringfield_S52	OBL	24	4	0.1573	1	1	244	CN52652_S52	UNK	11	5	0.1252	1	1
53	CN107801_S53	ROM	12	5	0.1502	1	1	149	AACHalli_S53	OBL	24	4	0.1372	1	1	245	CN52871_S53	RUS	2	5	0.1446	1	1
54	CN107803_S54	ROM	12	3	0.1530	1	1	150	AACShinju_S54	OBL	24	4	0.1541	1	1	246	CN52872_S54	CAN	1	5	0.1498	1	1
55	CN107805_S55	ROM	12	3	0.1311	1	1	151	AACRin_S55	OBL	24	4	0.1511	1	1	247	CN52873_S55	FRA	7	5	0.1267	1	1
56	CN107806_S56	SVK	23	5	0.1270	1	1	152	CN29751_S56	ROM	12	5	0.1716	1	1	248	CN52875_S56	NLD	13	5	0.1248	1	1
57	CN107807_S57	CAN	1	5	0.1403	1	1	153	CN30316_S57	ROM	12	5	0.1568	1	1	249	CN107406_S57	CHN	3	5	0.1331	1	1
58	CN107808_S58	SWE	4	3	0.1439	1	1	154	CN30637_S58	ITA	22	5	0.1533	1	1	250	CN107423_S58	CHN	3	5	0.1389	1	1
59	CN107809_S59	CAN	1	3	0.1485	1	1	155	CN30642_S59	DEU	6	5	0.1612	1	1	251	CN107449_S59	FRA	7	5	0.1228	1	1
60	CN107812_S60	CAN	1	2	0.1395	1	1	156	CN32320_S60	ITA	22	5	0.1608	1	1	252	CN107453_S60	FRA	7	5	0.1307	1	1
61	CN107817_S61	CAN	1	1	0.1464	1	1	157	CN32394_S61	POL	10	5	0.1585	1	1	253	CN107454_S61	SWE	4	5	0.1300	1	1
62	CN107818_S62	NLD	13	3	0.1495	1	1	158	CN32416_S62	AUT	20	5	0.1655	1	1	254	CN107462_S62	SWE	4	2	0.1318	1	1
63	CN107824_S63	BEL	14	1	0.1513	1	1	159	CN32662_S63	AUT	20	5	0.1660	1	1	255	CN107463_S63	JPN	8	2	0.1182	1	1
64	CN107827_S64	GBR	18	2	0.1402	1	1	160	CN32760_S64	DEU	6	5	0.1399	1	1	256	CN107464_S64	YUG	19	2	0.1471	1	1
65	CN107828_S65	BEL	14	2	0.1353	1	1	161	CN32766_S65	DEU	6	5	0.1680	1	1	257	CN107465_S65	RUS	2	2	0.1229	1	1
66	CN107833_S66	PRT	23	3	0.1498	1	1	162	CN33908_S66	DEU	6	5	0.1385	1	1	258	CN107466_S66	CHN	3	2	0.1297	1	1
67	CN107835_S67	FRA	7	3	0.1507	1	1	163	CN35313_S67	SRB	23	5	0.1598	1	1	259	CN107467_S67	BGR	21	2	0.1539	1	1
68	CN107837_S68	LTU	23	3	0.1466	1	1	164	CN29789_S68	BGR	21	5	0.1578	1	1	260	CN107468_S68	HUN	9	2	0.1262	1	1
69	CN107838_S69	BEL	14	3	0.1588	1	1	165	CN33913_S69	HUN	9	5	0.1514	1	1	261	CN107470_S69	CHN	3	2	0.1193	1	1
70	CN107839_S70	CHE	16	3	0.1658	1	1	166	CN35316_S70	HUN	9	5	0.1524	1	1	262	CN107471_S70	HUN	9	2	0.1247	1	1
71	CN107840_S71	NLD	13	3	0.1335	1	1	167	CN35320_S71	RUS	2	5	0.1458	1	1	263	CN107472_S71	RUS	2	2	0.1325	1	1
72	CN107842_S72	DEU	6	2	0.1459	1	1	168	CN35327_S72	RUS	2	5	0.1584	1	1	264	CN107474_S72	CHN	3	2	0.1492	1	1
73	CN107844_S73	NLD	13	2	0.1255	2	2	169	CN35340_S73	RUS	2	5	0.1143	2	2	265	CN107475_S73	NLD	13	2	0.1408	3	3
74	CN107845_S74	NLD	13	2	0.1390	2	2	170	CN35352_S74	BGR	21	5	0.1174	2	2	266	CN107481_S74	BEL	14	2	0.1306	3	3
75	CN107846_S75	NLD	13	2	0.1134	2	2	171	CN35358_S75	JPN	8	5	0.1179	2	2	267	CN107482_S75	BEL	14	2	0.1163	3	3
76	CN107847_S76	NLD	13	2	0.1208	2	2	172	CN35365_S76	RUS	2	5	0.1226	2	2	268	CN107483_S76	GBR	18	3	0.1192	3	3
77	CN107849_S77	NLD	13	2	0.1295	2	2	173	CN35370_S77	USA	15	5	0.1217	2	2	269	CN107484_S77	BEL	14	2	0.1111	3	3
78	CN107850_S78	NLD	13	2	0.1334	2	2	174	CN35377_S78	CHN	3	5	0.1161	2	2	270	CN107485_S78	SWE	4	2	0.1165	3	3
79	CN107852_S79	NLD	13	1	0.1168	2	2	175	CN36219_S79	JPN	8	5	0.1134	2	2	271	CN107487_S79	SWE	4	2	0.1237	3	3
80	CN107853_S80	SWE	4	2	0.1174	2	2	176	CN36333_S80	JPN	8	5	0.1148	2	2	272	CN107488_S80	SWE	4	2	0.1216	3	3
81	CN107855_S81	AUT	20	2	0.1047	2	2	177	CN36339_S81	JPN	8	5	0.1235	2	2	273	CN107489_S81	SWE	4	2	0.1447	3	3
82	CN107857_S82	DEU	6	5	0.1426	2	2	178	CN39090_S82	JPN	8	5	0.1272	2	2	274	CN107491_S82	SWE	4	2	0.1111	3	3
83	CN107862_S83	DEU	6	2	0.1262	2	2	179	CN39153_S83	JPN	8	5	0.1104	2	2	275	CN107500_S83	SWE	4	1	0.1253	3	3
84	CN107864_S84	FRA	7	2	0.1184	2	2	180	CN39155_S84	POL	10	5	0.1085	2	2	276	CN107502_S84	DEU	6	3	0.1960	3	3
85	CN107865_S85	FRA	7	1	0.1137	2	2	181	CN39162_S85	POL	10	5	0.1090	2	2	277	CN107503_S85	DEU	6	2	0.1342	3	3
86	CN107866_S86	FRA	7	2	0.1249	2	2	182	CN39173_S86	POL	10	5	0.1065	2	2	278	CN107506_S86	YUG	19	2	0.1260	3	3
87	CN107867_S87	FRA	7	2	0.1289	2	2	183	CN39187_S87	POL	10	5	0.1083	2	2	279	CN107507_S87	ROM	12	2	0.1097	3	3
88	CN107871_S88	FRA	7	2	0.1202	2	2	184	CN39215_S88	POL	10	5	0.1078	2	2	280	CN107509_S88	POL	10	2	0.1113	3	3
89	CN107872_S89	FRA	7	2	0.1250	2	2	185	CN42388_S89	POL	10	5	0.1081	2	2	281	CN107515_S89	SWE	4	1	0.1194	3	3
90	CN107873_S90	SWE	4	1	0.1164	2	2	186	CN42391_S90	POL	10	5	0.1154	2	2	282	CN107521_S90	POL	10	2	0.1127	3	3
91	CN107874_S91	SWE	4	2	0.1267	2	2	187	CN42535_S91	JPN	8	5	0.1162	2	2	283	CN107525_S91	POL	10	1	0.1279	3	3
92	CN107875_S92	SWE	4	2	0.1305	2	2	188	CN44031_S92	JPN	8	5	0.1216	2	2	284	CN107535_S92	POL	10	1	0.1181	3	3
93	CN107876_S93	SWE	4	1	0.1164	2	2	189	CN45092_S93	UNK	11	5	0.1210	2	2	285	CN107538_S93	POL	10	2	0.1049	3	3
94	CN107877_S94	SWE	4	1	0.1130	2	2	190	CN52632_S94	UNK	11	5	0.1144	2	2	286	CN107540_S94	SWE	4	1	0.1252	3	3
95	CN107878_S95	SWE	4	2	0.1259	2	2	191	CN107493_S95	UNK	11	2	0.1147	2	2	287	CN107541_S95	UNK	11	1	0.1221	3	3
96	XCN35382_S96							192	X1CN35382_S96							288	CN107543_S96	BEL	14	2	0.1163	3	3

289	CN30629_S1	ROM	12	5	0.1274	2	2	385	CN30318_S1	CHN	3	5	0.1269	2	2	481	CN35332_S1	KOR	5	5	0.1188	2	2
290	CN32352_S2	KOR	5	5	0.2522	2	2	386	CN30644_S2	HUN	9	5	0.1340	2	2	482	CN35341_S2	KOR	5	5	0.1180	2	2
291	CN32631_S3	KOR	5	5	0.1094	2	2	387	CN31631_S3	ROM	12	5	0.1118	2	2	483	CN39109_S3	UNK	11	5	0.1150	2	2
292	CN32669_S4	CAN	1	5	0.1153	2	2	388	CN31719_S4	NLD	13	5	0.1294	2	2	484	CN107461_S4	CHN	3	5	0.1165	2	2
293	CN35310_S5	CAN	1	5	0.1147	2	2	389	CN31984_S5	NOR	23	5	0.1225	2	2	485	CN107665_S5	RUS	2	5	0.1158	2	2
294	CN35311_S6	CAN	1	5	0.1172	2	2	390	CN32354_S6	HUN	9	5	0.1129	2	2	486	CN107810_S6	FRA	7	3	0.1088	2	2
295	CN35344_S7	CHN	3	5	0.1290	2	2	391	CN35337_S7	KOR	5	5	0.1194	2	2	487	CN107811_S7	FRA	7	2	0.1181	2	2
296	CN39071_S8	FRA	7	5	0.1208	2	2	392	CN35342_S8	KOR	5	5	0.1278	2	2	488	CN107813_S8	SWE	4	2	0.1047	2	2
297	CN39168_S9	DEU	6	5	0.1118	2	2	393	CN35383_S9	KOR	5	5	0.1216	2	2	489	CN107814_S9	SWE	4	2	0.1113	2	2
298	CN52636_S10	UNK	11	5	0.0986	2	2	394	CN36009_S10	CHN	3	5	0.1200	2	2	490	CN107815_S10	SWE	4	2	0.1149	2	2
299	CN52644_S11	KOR	5	5	0.1136	2	2	395	CN36218_S11	CHN	3	5	0.1061	2	2	491	CN107816_S11	SWE	4	2	0.1146	2	2
300	CN52651_S12	SWE	4	5	0.1157	2	2	396	CN52628_S12	SWE	4	1	0.1228	2	2	492	CN107819_S12	JPN	8	3	0.1197	2	2
301	CN52653_S13	FRA	7	5	0.1115	2	2	397	CN107473_S13	BEL	14	2	0.1173	2	2	493	CN107820_S13	SWE	4	1	0.1164	2	2
302	CN107424_S14	FRA	7	5	0.1105	2	2	398	CN107512_S14	FRA	7	2	0.1202	2	2	494	CN107826_S14	RUS	2	2	0.1144	2	2
303	CN107513_S15	SWE	4	2	0.1129	2	2	399	CN107592_S15	JPN	8	2	0.1199	2	2	495	CN107830_S15	HUN	9	2	0.1156	2	2
304	CN107531_S16	CHN	3	2	0.1164	2	2	400	CN107602_S16	ROM	12	2	0.1234	2	2	496	CN107832_S16	POL	10	2	0.1197	2	2
305	CN107544_S17	RUS	2	1	0.1095	2	2	401	CN107831_S17	POL	10	1	0.1176	2	2	497	CN107836_S17	HUN	9	3	0.1150	2	2
306	CN107545_S18	RUS	2	2	0.1077	2	2	402	CN107834_S18	HUN	9	3	0.1233	2	2	498	CN107848_S18	SWE	4	2	0.1291	2	2
307	CN107546_S19	RUS	2	2	0.1067	2	2	403	CN35323_S19	KOR	5	5	0.1113	2	2	499	CN107856_S19	SWE	4	2	0.1243	2	2
308	CN107547_S20	RUS	2	2	0.1132	2	2	404	CN35333_S20	KOR	5	5	0.1140	2	2	500	CN107858_S20	SWE	4	2	0.1138	2	2
309	CN107548_S21	RUS	2	1	0.1095	2	2	405	CN35334_S21	KOR	5	5	0.1262	2	2	501	CN107860_S21	JPN	8	1	0.1446	2	2
310	CN107552_S22	RUS	2	1	0.1093	2	2	406	CN35343_S22	KOR	5	5	0.1192	2	2	502	CN107861_S22	SWE	4	1	0.1165	2	2
311	CN107553_S23	RUS	2	2	0.1113	2	2	407	CN35354_S23	KOR	5	5	0.1153	2	2	503	CN107870_S23	DEU	6	2	0.1187	2	2
312	CN107554_S24	RUS	2	2	0.1152	2	2	408	CN35356_S24	KOR	5	5	0.1181	2	2	504	CN29742_S24	CHN	3	5	0.1139	2	2
313	CN107555_S25	RUS	2	3	0.1035	2	2	409	CN35357_S25	KOR	5	5	0.1448	3	3	505	CN29747_S25	CHN	3	5	0.1510	3	3
314	CN107556_S26	CAN	1	2	0.1239	2	2	410	CN35361_S26	KOR	5	5	0.1478	3	3	506	CN29749_S26	CHN	3	5	0.1478	3	3
315	CN107558_S27	CAN	1	3	0.1077	2	2	411	CN35756_S27	RUS	2	5	0.1311	3	3	507	CN29750_S27	CHN	3	5	0.1401	3	3
316	CN107560_S28	CAN	1	2	0.1293	2	2	412	CN35917_S28	RUS	2	5	0.1472	3	3	508	CN29790_S28	CHN	3	5	0.1438	3	3
317	CN107561_S29	CAN	1	2	0.1134	2	2	413	CN36008_S29	CHN	3	5	0.1111	3	3	509	CN29798_S29	CHN	3	5	0.1510	3	3
318	CN107562_S30	CAN	1	2	0.1192	2	2	414	CN36214_S30	CHN	3	5	0.1328	3	3	510	CN32001_S30	UNK	11	5	0.1347	3	3
319	CN107563_S31	CAN	1	1	0.1219	2	2	415	CN36216_S31	CHN	3	5	0.1414	3	3	511	CN33247_S31	CAN	1	5	0.1314	3	3
320	CN107568_S32	CAN	1	2	0.1255	2	2	416	CN36217_S32	CHN	3	5	0.1392	3	3	512	CN33251_S32	CAN	1	5	0.1311	3	3
321	CN107566_S33	CAN	1	2	0.1040	2	2	417	CN36336_S33	RUS	2	5	0.1560	3	3	513	CN33254_S33	UNK	11	5	0.1368	3	3
322	CN107569_S34	CAN	1	3	0.1138	2	2	418	CN39077_S34	CAN	1	5	0.1597	3	3	514	CN33257_S34	CAN	1	5	0.1504	3	3
323	CN107571_S35	CAN	1	3	0.1032	2	2	419	CN39084_S35	CAN	1	5	0.1699	3	3	515	CN33258_S35	CAN	1	5	0.1365	3	3
324	CN107572_S36	CAN	1	3	0.1054	2	2	420	CN39086_S36	CAN	1	5	0.1515	3	3	516	CN33260_S36	CAN	1	5	0.1386	3	3
325	CN107573_S37	CAN	1	2	0.1138	2	2	421	CN39087_S37	CAN	1	5	0.1405	3	3	517	CN33263_S37	CAN	1	5	0.1324	3	3
326	CN107574_S38	CAN	1	3	0.1143	2	2	422	CN39096_S38	CAN	1	5	0.1268	3	3	518	CN33264_S38	CAN	1	5	0.1269	3	3
327	CN107577_S39	CAN	1	2	0.0998	2	2	423	CN39099_S39	CAN	1	5	0.1337	3	3	519	CN33266_S39	CAN	1	5	0.1351	3	3
328	CN107578_S40	CAN	1	1	0.1282	2	2	424	CN39114_S40	CAN	1	5	0.1344	3	3	520	CN33267_S40	CAN	1	5	0.1294	3	3
329	CN107579_S41	CAN	1	2	0.1024	2	2	425	CN39131_S41	CAN	1	5	0.1473	3	3	521	CN33277_S41	CAN	1	5	0.1345	3	3
330	CN107580_S42	CAN	1	2	0.1062	2	2	426	CN39150_S42	CAN	1	5	0.1347	3	3	522	CN33278_S42	CAN	1	5	0.1363	3	3
331	CN107581_S43	CAN	1	2	0.1042	2	2	427	CN39169_S43	CAN	1	5	0.1322	3	3	523	CN33279_S43	CAN	1	5	0.1369	3	3
332	CN107582_S44	CAN	1	2	0.1170	2	2	428	CN39193_S44	CAN	1	5	0.1341	3	3	524	CN35264_S44	UNK	11	5	0.1337	3	3
333	CN107583_S45	CAN	1	2	0.1311	2	2	429	CN42536_S45	CHN	3	5	0.1415	3	3	525	CN35265_S45	UNK	11	5	0.1383	3	3
334	CN107585_S46	CAN	1	2	0.1062	2	2	430	CN51844_S46	CAN	1	5	0.1218	3	3	526	CN35319_S46	KOR	5	5	0.1464	3	3
335	CN107588_S47	CAN	1	2	0.1046	2	2	431	CN51845_S47	CAN	1	5	0.1319	3	3	527	CN35330_S47	KOR	5	5	0.1439	3	3
336	CN107593_S48	CAN	1	2	0.1143	2	2	432	CN107353_S48	CAN	1	5	0.1331	3	3	528	CN35331_S48	KOR	5	5	0.1451	3	3
337	CN107594_S49	CAN	1	1	0.1195	1	1	433	CN107385_S49	CAN	1	5	0.1465	1	1	529	CN35339_S49	KOR	5	5	0.1710	1	1
338	CN107595_S50	CAN	1	1	0.1346	1	1	434	CN107397_S50	CAN	1	5	0.1556	1	1	530	CN35364_S50	KOR	5	5	0.1638	1	1
339	CN107596_S51	CAN	1	1	0.1513	1	1	435	CN107408_S51	CAN	1	5	0.1556	1	1	531	CN36135_S51	CAN	1	5	0.1538	1	1
340	CN107597_S52	CAN	1	1	0.1221	1	1	436	CN107476_S52	HUN	9	2	0.1786	1	1	532	CN36215_S52	CHN	3	5	0.1540	1	1
341	CN107599_S53	CAN	1	2	0.1491	1	1	437	CN107480_S53	FRA	7	2	0.1542	1	1	533	CN36249_S53	UNK	11	5	0.1478	1	1
342	CN107600_S54	CAN	1	2	0.1450	1	1	438	CN107490_S54	DEU	6	2	0.1510	1	1	534	CN39075_S54	UNK	11	5	0.1664	1	1
343	CN107603_S55	CAN	1	2	0.1273	1	1	439	CN107492_S55	DEU	6	2	0.1509	1	1	535	CN39178_S55	CAN	1	5	0.1554	1	1
344	CN107604_S56	CHN	3	3	0.1287	1	1	440	CN107494_S56	DEU	6	2	0.1565	1	1	536	CN42220_S56	CAN	1	5	0.1550	1	1
345	CN107605_S57	CHN	3	2	0.1275	1	1	441	CN107495_S57	DEU	6	2	0.1767	1	1	537	CN42379_S57	CAN	1	5	0.1495	1	1
346	CN107606_S58	RUS	2	2	0.1420	1	1	442	CN107496_S58	DEU	6	2	0.1685	1	1	538	CN42387_S58	CAN	1	5	0.1479	1	1
347	CN107608_S59	HUN	9	1	0.1172	1	1	443	CN107497_S59	DEU	6	1	0.1672	1	1	539	CN45056_S59	CAN	1	5	0.1527	1	1
348	CN107609_S60	DEU	6	3	0.1329	1	1	444	CN107498_S60	DEU	6	1	0.1750	1	1	540	CN46335_S60	CAN	1	5	0.1463	1	1
349	CN107610_S61	DEU	6	2	0.1441	1	1	445	CN107510_S61	DEU	6	2	0.1461	1	1	541	CN51846_S61	CAN	1	5	0.1450	1	1
350	CN107611_S62	DEU	6	2	0.1348	1	1	446	CN107514_S62	FRA	7	2	0.1775	1	1	542	CN52740_S62	CAN	1	5	0.1349	1	1
351	CN107613_S63	DEU	6	2	0.1257	1	1	447	CN107516_S63	FRA	7	1	0.1531	1	1	543	CN52874_S63	UNK	11	5	0.1558	1	1
352	CN107615_S64	POL	10	1	0.1296	1	1	448	CN107517_S64	FRA	7	2	0.1551	1	1	544	CN52877_S64	UNK	11	5	0.1693	1	1
353	CN107616_S65	POL	10	2	0.1320	1	1	449	CN107518_S65	FRA	7	2	0.1603	1	1	545	CN107352_S65	CAN	1	5	0.1537	1	1
354	CN107617_S66	CHN	3	2	0.1238	1	1	450	CN107519_S66	FRA	7	2	0.1511	1	1	546	CN107359_S66	CAN	1	5	0.1515	1	1
355	CN107618_S67	KOR	5	2	0.1442	1	1	451	CN107520_S67	SWE	4	2	0.1468	1	1	547	CN107360_S67	CAN	1	5	0.1583	1	1
356	CN107619_S68	CHN	3	2	0.1276	1	1	452	CN107522_S68	SWE	4	2	0.1694	1	1	548	CN107361_S68	CAN	1	5	0.1504	1	1
357	CN107620_S69	CHN	3	2	0.1199	1	1	453	CN107523_S69	SWE	4	1	0.1590	1	1	549	CN107370_S69	CAN	1	5	0.1378	1	1
358	CN107621_S70	KOR	5	2	0.1231	1	1	454	CN107526_S70	SWE	4	2	0.1523	1	1	550	CN107373_S70	CAN	1	5	0.1503	1	1
359	CN107622_S71	KOR	5	3	0.1270	1	1	455	CN107527_S71	SWE	4	1	0.1588	1	1	551	CN107375_S71	CAN	1	5	0.1460	1	1
360	CN107623_S72	KOR	5	2	0.1499	1	1	456	CN107530_S72	SWE	4	2	0.1499	1	1	552	CN107389_S72	CAN	1	5	0.1415	1	1
361	CN107624_S73	KOR	5	2	0.1069	3	3	457	CN107532_S73	SWE	4	2	0.1205	2	2	553	CN107391_S73	CAN	1	5	0.1134	2	2
362	CN107625_S74	KOR	5	1	0.1100	3	2	458	CN107534_S74	SWE	4	1	0.1241	2	2	554	CN107394_S74	CAN	1	5	0.1109	2	2
363	CN107626_S75	KOR	5	2	0.1117	3	3	459	CN107536_S75	SWE	4	1	0.1341	2	2	555	CN107395_S75	CAN	1	5	0.1080	2	2
364	CN107627_S76	KOR	5	2	0.1112	3	3	460	CN107542_S76	SWE	4	1	0.1373	2	2	556	CN107403_S76	CAN	1	5	0.1144	2	2
365	CN107628_S77	KOR	5	2	0.1205	3	3	461	CN107549_S77	DEU	6	1	0.1115	2	2	557	CN107405_S77	CAN	1	5	0.1113	2	2
366	CN107629_S78	KOR	5	1	0.1118	3	3	462	CN107550_S78	DEU	6	1	0.1075	2	2	558	CN107407_S78	CAN	1	5	0.1092	2	2
367	CN107632_S79	CHN	3	2	0.1328	3	3	463	CN107557_S79	HUN	9	3	0.1436	2	2	559	CN107413_S79	CAN	1	5	0.1128	2	2
368	CN107634_S80	RUS	2	2	0.1172	3	3	464	CN107559_S80	HUN	9	2	0.1183	2	2	560	CN107420_S80	CAN	1	5	0.1168	2	2
369	CN107635_S81	CHN	3	2	0.1174	3	3	465	CN107565_S81	CHN	3	2	0.1191	2	2	561	CN107421_S81	CAN	1	5	0.1063	2	2
370	CN107636_S82	CAN	1	1	0.1171	3	3	466	CN107567_S82	RUS	2	2	0.1206	2	2	562	CN107422_S82	CAN	1	5	0.1067	2	2
371	CN107637_S83	CAN	1	2	0.1223	3	3	467	CN107575_S83	CHN	3	3	0.0999	2	2	563	CN107425_S83	CAN	1	5	0.1052	2	2
372	CN107638_S84	CAN	1	2	0.1204	3	3	468	CN107576_S84	CHN	3	2	0.1115	2	2	564	CN107436_S84	CAN	1	5	0.1151	2	2
373	CN107639_S85	CAN	1	1	0.1178	3	3	469	CN107586_S85	CHN	3	2	0.1081	2	2	565	CN107438_S85	CAN	1	5	0.1118	2	2
374	CN107640_S86	CAN	1	2	0.1223	3	3	470	CN107587_S86	CHN	3	2	0.1135	2	2	566	CN107439_S86	CAN	1	5	0.1123	2	2
375	CN107641_S87	CAN	1	1	0.1211	3	3	471	CN107591_S87	SWE	4	1	0.1199	2	2	567	CN107443_S87	CAN	1	5	0.1204	2	2
376	CN107642_S88	CAN	1	2	0.1127	3	3	472	CN107601_S88	DEU	6	1	0.1229	2	2	568	CN107447_S88	CAN	1	5	0.1135	2	2
377	CN107643_S89	CAN	1	2	0.1215	3	3	473	CN107607_S89	RUS	2	2	0.1090	2	2	569	CN107451_S89	CAN	1	5	0.1136	2	2
378	CN107645_S90	CAN	1	1	0.1347	3	3	474	CN107612_S90	DEU	6	2	0.1191	2	2	570	CN107459_S90	CAN	1	5	0.1183	2	2
379	CN107646_S91	CHN	3	2	0.1113	3	3	475	CN107614_S91	DEU	6	1	0.1054	2	2	571	CN107469_S91	CAN	1	2	0.1214	2	2
380	CN107647_S92	FRA	7	5	0.1284	3	3	476	CN107631_S92	JPN	8	2	0.1173	2	2	572	CN107570_S92	CHN	3	2	0.1170	2	2
381	CN107648_S93	FRA	7	5	0.1181	3	3	477	CN107633_S93	JPN	8	2	0.1136	2	2	573	CN107589_S93	CHN	3	2	0.1107	2	2
382	CN107649_S94	RUS	2	5	0.1145	3	3	478	CN107644_S94	POL	10	2	0.1075	2	2	574	CN107802_S94	CAN	1	5	0.1300	2	2
383	CN107651_S95	DEU	6	5	0.1098	3	3	479	CN107653_S95	POL	10	5	0.1267	2	2	575	CN107825_S95	CHN	3	3	0.1241	2	2
384	X2CN35382_S96							480	X3CN35382_S96							576	X4CN35382_S96						

Note: Sample was labeled with S1 to S96 after an accession (or breeding line) in each of six sequencing runs and the sample CN35382 was repeated in the other runs for quality control. The ISO 639-2 code is used for a country. UNK is for unknown and OBL is for AAFC Ottawa breeding line. MG 1 is for MG 000, 2 for MG 00, 3 for MG 0, 4 for OBL and 5 for the rest. Clusters from PCoA and Structure are shown.

Table S3. Sequence summary for 571 assayed soybean samples, including NCBI accession information.

Key	NCBI accession	Sample	Raw reads	Filtered reads	Key	NCBI accession	Sample	Raw reads	Filtered reads	Key	NCBI accession	Sample	Raw reads	Filtered reads
1	SAMN18102822	CN29752	1,191,331	1,070,473	97	SAMN18102918	CN32351	1,325,514	1,182,712	193	SAMN18103014	CN30633	890,153	753,935
2	SAMN18102823	CN29792	1,121,537	1,042,152	98	SAMN18102919	CN35348	1,117,338	1,000,042	194	SAMN18103015	CN30634	802,154	664,427
3	SAMN18102824	CN29793	356,138	335,707	99	SAMN18102920	CN35373	1,102,693	985,011	195	SAMN18103016	CN31690	786,684	666,546
4	SAMN18102825	CN29795	1,106,121	1,010,604	100	SAMN18102921	CN42821	1,147,578	1,032,452	196	SAMN18103017	CN31692	856,358	748,875
5	SAMN18102826	CN30389	1,037,252	951,490	101	SAMN18102922	CN107435	1,157,584	1,040,328	197	SAMN18103018	CN32053	902,588	749,199
6	SAMN18102827	CN30391	1,000,473	911,911	102	SAMN18102923	CN107437	1,071,745	976,345	198	SAMN18103019	CN32257	1,089,948	921,100
7	SAMN18102828	CN32356	957,516	871,907	103	SAMN18102924	CN107650	1,083,639	976,602	199	SAMN18103020	CN32442	1,024,226	862,787
8	SAMN18102829	CN32451	1,169,603	1,065,773	104	SAMN18102925	CN107804	995,603	888,347	200	SAMN18103021	CN32634	1,246,185	1,063,076
9	SAMN18102830	CN32547	1,048,690	952,543	105	SAMN18102926	CN107822	1,083,999	966,479	201	SAMN18103022	CN32639	797,073	633,001
10	SAMN18102831	CN32826	964,792	902,287	106	SAMN18102927	CN107879	967,479	866,372	202	SAMN18103023	CN32829	852,715	734,979
11	SAMN18102832	CN33248	903,496	825,280	107	SAMN18102928	CN107880	894,686	804,226	203	SAMN18103024	CN33253	676,143	582,483
12	SAMN18102833	CN33259	957,876	873,373	108	SAMN18102929	CN107881	909,461	823,434	204	SAMN18103025	CN33255	792,137	686,637
13	SAMN18102834	CN35309	1,056,761	963,361	109	SAMN18102930	CN107882	798,377	725,717	205	SAMN18103026	CN33265	725,701	610,250
14	SAMN18102835	CN35312	835,423	766,561	110	SAMN18102931	CN107883	811,422	736,665	206	SAMN18103027	CN33273	720,564	627,058
15	SAMN18102836	CN35314	839,682	778,119	111	SAMN18102932	CN107884	792,812	719,124	207	SAMN18103028	CN33911	609,711	517,658
16	SAMN18102837	CN35321	854,876	777,491	112	SAMN18102933	CN107885	894,223	829,646	208	SAMN18103029	CN35308	876,285	747,841
17	SAMN18102838	CN35329	970,524	882,728	113	SAMN18102934	CN115251	826,076	759,882	209	SAMN18103030	CN35318	975,988	821,291
18	SAMN18102839	CN35380	944,423	877,478	114	SAMN18102935	CN115252	879,542	815,274	210	SAMN18103031	CN35372	832,872	689,982
19	SAMN18102840	CN35919	1,201,627	1,131,061	115	SAMN18102936	CN115253	706,229	648,707	211	SAMN18103032	CN35382	889,201	747,163
20	SAMN18102841	CN36138	875,212	799,466	116	SAMN18102937	CN115254	839,645	769,492	212	SAMN18103033	CN35757	872,912	729,298
21	SAMN18102842	CN36143	955,699	872,599	117	SAMN18102938	CN115255	813,459	756,687	213	SAMN18103034	CN35758	667,575	521,718
22	SAMN18102843	CN36334	324,339	295,518	118	SAMN18102939	CN115256	907,745	836,164	214	SAMN18103035	CN35916	821,527	681,858
23	SAMN18102844	CN52642	838,926	769,149	119	SAMN18102940	CN115257	885,542	812,728	215	SAMN18103036	CN35918	764,824	666,470
24	SAMN18102845	CN107446	874,132	803,280	120	SAMN18102941	CN115258	724,185	664,886	216	SAMN18103037	CN35920	806,116	689,433
25	SAMN18102846	CN107450	1,129,720	1,062,994	121	SAMN18102942	CN115259	1,076,671	1,002,120	217	SAMN18103038	CN35921	941,324	884,267
26	SAMN18102847	CN107455	1,068,991	1,009,178	122	SAMN18102943	Maple Arrow	1,043,797	972,858	218	SAMN18103039	CN36139	941,521	888,271
27	SAMN18102848	CN107477	989,394	933,369	123	SAMN18102944	Maple Glen	1,001,205	930,962	219	SAMN18103040	CN36141	840,749	793,152
28	SAMN18102849	CN107479	889,827	837,541	124	SAMN18102945	AC Orford	931,195	868,065	220	SAMN18103041	CN36335	845,629	797,358
29	SAMN18102850	CN107486	1,118,240	1,052,321	125	SAMN18102946	Accord	1,069,688	998,562	221	SAMN18103042	CN36337	954,298	903,432
30	SAMN18102851	CN107501	957,545	909,857	126	SAMN18102947	RD714	376,191	353,552	222	SAMN18103043	CN36340	956,668	906,262
31	SAMN18102852	CN107511	891,273	839,863	127	SAMN18102948	AC Glengarry	986,797	920,319	223	SAMN18103044	CN36342	914,354	863,921
32	SAMN18102853	CN107524	992,063	933,858	128	SAMN18102949	DH3604	1,076,459	1,004,811	224	SAMN18103045	CN39070	976,468	920,752
33	SAMN18102854	CN107529	957,417	902,303	129	SAMN18102950	Toki	1,091,754	1,015,549	225	SAMN18103046	CN42928	1,009,845	954,816
34	SAMN18102855	CN107590	1,173,594	1,108,886	130	SAMN18102951	Chikala	951,741	892,072	226	SAMN18103047	CN45087	911,196	859,376
35	SAMN18102856	CN107598	1,155,661	1,085,400	131	SAMN18102952	QGC10N	954,420	889,274	227	SAMN18103048	CN45088	914,203	859,624
36	SAMN18102857	CN107630	1,011,937	954,274	132	SAMN18102953	QGC12N	939,707	877,730	228	SAMN18103049	CN45090	906,495	857,741
37	SAMN18102858	CN107652	1,057,593	999,377	133	SAMN18102954	Dares	809,331	753,186	229	SAMN18103050	CN45095	983,076	932,182
38	SAMN18102859	CN107654	969,074	913,382	134	SAMN18102955	Apalis	881,044	822,470	230	SAMN18103051	CN45107	974,050	925,470
39	SAMN18102860	CN107655	962,238	905,063	135	SAMN18102956	Loriot	829,932	773,796	231	SAMN18103052	CN51375	1,084,696	1,019,326
40	SAMN18102861	CN107656	1,009,650	951,244	136	SAMN18102957	Jari	1,044,801	976,711	232	SAMN18103053	CN52633	1,136,367	1,075,223
41	SAMN18102862	CN107657	924,131	869,993	137	SAMN18102958	DH710	1,048,318	976,046	233	SAMN18103054	CN52634	587,744	557,582
42	SAMN18102863	CN107658	927,837	875,026	138	SAMN18102959	SG1010	795,003	741,824	234	SAMN18103055	CN52635	844,346	801,622
43	SAMN18102864	CN107659	1,039,282	977,723	139	SAMN18102960	Roselin	822,841	767,676	235	SAMN18103056	CN52639	1,001,946	944,261
44	SAMN18102865	CN107660	912,379	860,169	140	SAMN18102961	AAC Vireo	860,016	800,879	236	SAMN18103057	CN52640	965,351	903,821
45	SAMN18102866	CN107661	920,897	871,254	141	SAMN18102962	AAC Edward	917,208	855,594	237	SAMN18103058	CN52643	905,735	856,881
46	SAMN18102867	CN107662	977,450	919,349	142	SAMN18102963	AAC Naruto	833,044	780,056	238	SAMN18103059	CN52645	927,249	875,525
47	SAMN18102868	CN107663	1,155,572	1,087,868	143	SAMN18102964	Heron	802,484	752,202	239	SAMN18103060	CN52646	878,060	827,698
48	SAMN18102869	CN107664	820,310	774,117	144	SAMN18102965	AC Colibri	1,097,186	1,034,276	240	SAMN18103061	CN52647	995,411	939,599
49	SAMN18102870	CN107666	1,061,151	988,732	145	SAMN18102966	Nattosan	921,782	858,666	241	SAMN18103062	CN52648	867,366	825,924
50	SAMN18102871	CN107667	993,828	928,331	146	SAMN18102967	Canatto	941,142	874,554	242	SAMN18103063	CN52649	931,532	884,961
51	SAMN18102872	CN107668	958,580	904,290	147	SAMN18102968	AAC Umami	849,276	789,215	243	SAMN18103064	CN52650	876,745	833,660
52	SAMN18102873	CN107800	870,696	814,130	148	SAMN18102969	AAC Springfield	878,111	815,256	244	SAMN18103065	CN52652	783,150	744,428
53	SAMN18102874	CN107801	930,016	869,795	149	SAMN18102970	AAC Halli	1,055,548	987,946	245	SAMN18103066	CN52871	925,758	874,555
54	SAMN18102875	CN107803	911,939	854,217	150	SAMN18102971	AAC Shinju	1,022,585	954,483	246	SAMN18103067	CN52872	922,634	875,191
55	SAMN18102876	CN107805	983,437	919,611	151	SAMN18102972	AAC Rin	1,073,546	1,004,745	247	SAMN18103068	CN52873	621,259	591,589
56	SAMN18102877	CN107806	988,495	930,265	152	SAMN18102973	CN29751	1,178,062	1,095,622	248	SAMN18103069	CN52875	822,537	784,309
57	SAMN18102878	CN107807	966,970	901,752	153	SAMN18102974	CN30316	1,039,389	970,373	249	SAMN18103070	CN107406	996,276	942,631
58	SAMN18102879	CN107808	994,952	929,291	154	SAMN18102975	CN30637	937,383	875,721	250	SAMN18103071	CN107423	901,893	852,894
59	SAMN18102880	CN107809	1,217,471	1,137,806	155	SAMN18102976	CN30642	988,843	923,393	251	SAMN18103072	CN107449	419,920	400,330
60	SAMN18102881	CN107812	1,110,699	1,036,223	156	SAMN18102977	CN32320	978,759	912,169	252	SAMN18103073	CN107453	638,331	607,742
61	SAMN18102882	CN107817	1,043,445	976,231	157	SAMN18102978	CN32394	1,019,960	948,491	253	SAMN18103074	CN107454	976,781	926,753
62	SAMN18102883	CN107818	920,062	859,122	158	SAMN18102979	CN32416	1,015,942	944,513	254	SAMN18103075	CN107462	980,606	932,076
63	SAMN18102884	CN107824	980,421	913,593	159	SAMN18102980	CN32662	982,088	911,688	255	SAMN18103076	CN107463	496,829	473,271
64	SAMN18102885	CN107827	1,244,540	1,168,243	160	SAMN18102981	CN32760	574,988	537,555	256	SAMN18103077	CN107464	1,064,090	1,006,958
65	SAMN18102886	CN107828	982,086	919,334	161	SAMN18102982	CN32766	1,026,271	953,361	257	SAMN18103078	CN107465	737,506	702,551
66	SAMN18102887	CN107833	912,161	851,552	162	SAMN18102983	CN33908	949,860	887,604	258	SAMN18103079	CN107466	945,722	898,714
67	SAMN18102888	CN107835	1,059,970	994,604	163	SAMN18102984	CN35313	898,595	836,255	259	SAMN18103080	CN107467	986,078	933,056
68	SAMN18102889	CN107837	765,297	712,745	164	SAMN18102985	CN29789	938,963	872,021	260	SAMN18103081	CN107468	800,103	761,520
69	SAMN18102890	CN107838	975,711	912,238	165	SAMN18102986	CN33913	1,183,278	1,106,551	261	SAMN18103082	CN107470	431,465	411,012
70	SAMN18102891	CN107839	1,099,685	1,027,144	166	SAMN18102987	CN35316	1,102,267	1,034,426	262	SAMN18103083	CN107471	791,697	753,210
71	SAMN18102892	CN107840	930,034	872,237	167	SAMN18102988	CN35320	1,167,742	1,092,153	263	SAMN18103084	CN107472	740,599	702,963
72	SAMN18102893	CN107842	1,064,015	994,329	168	SAMN18102989	CN35327	1,134,881	1,058,539	264	SAMN18103085	CN107474	852,684	804,191
73	SAMN18102894	CN107844	985,759	926,687	169	SAMN18102990	CN35340	1,102,968	1,033,474	265	SAMN18103086	CN107475	989,665	935,112
74	SAMN18102895	CN107845	1,165,004	1,094,234	170	SAMN18102991	CN35352	1,075,718	1,009,169	266	SAMN18103087	CN107481	892,591	845,181
75	SAMN18102896	CN107846	968,070	912,317	171	SAMN18102992	CN35358	1,068,991	1,005,192	267	SAMN18103088	CN107482	520,115	495,744
76	SAMN18102897	CN107847	903,034	849,970	172	SAMN18102993	CN35365	1,006,836	943,834	268	SAMN18103089	CN107483	871,839	826,496
77	SAMN18102898	CN107849	1,012,979	952,426	173	SAMN18102994	CN35370	970,538	910,076	269	SAMN18103090	CN107484	602,878	573,924
78	SAMN18102899	CN107850	896,886	842,845	174	SAMN18102995	CN35377	913,937	855,996	270	SAMN18103091	CN107485	776,048	737,373
79	SAMN18102900	CN107852	924,710	873,708	175	SAMN18102996	CN36219	887,034	830,420	271	SAMN18103092	CN107487	832,591	788,532
80	SAMN18102901	CN107853	987,538	926,320	176	SAMN18102997	CN36333	1,031,841	968,016	272	SAMN18103093	CN107488	852,124	809,768
81	SAMN18102902	CN107855	1,074,236	1,016,511	177	SAMN18102998	CN36339	1,027,431	959,572	273	SAMN18103094	CN107489	973,226	923,680
82	SAMN18102903	CN107857	1,039,871	982,096	178	SAMN18102999	CN39090	990,724	927,587	274	SAMN18103095	CN107491	401,154	381,463
83	SAMN18102904	CN107862	974,586	918,125	179	SAMN18103000	CN39153	941,873	882,354	275	SAMN18103096	CN107500	1,010,197	956,016
84	SAMN18102905	CN107864	973,091	915,195	180	SAMN18103001	CN39155	959,889	898,627	276	SAMN18103097	CN107502	917,647	870,412
85	SAMN18102906	CN107865	1,033,267	966,646	181	SAMN18103002	CN39162	948,253	886,444	277	SAMN18103098	CN107503	985,652	936,696
86	SAMN18102907	CN107866	1,109,749	1,041,870	182	SAMN18103003	CN39173	941,954	882,852	278	SAMN18103099	CN107506	1,133,035	1,075,416
87	SAMN18102908	CN107867	958,072	897,930	183	SAMN18103004	CN39187	907,408	847,557	279	SAMN18103100	CN107507	663,976	630,985
88	SAMN18102909	CN107871	1,009,682	947,632	184	SAMN18103005	CN39215	1,140,323	1,067,820	280	SAMN18103101	CN107509	1,102,103	1,047,264
89	SAMN18102910	CN107872	986,378	926,016	185	SAMN18103006	CN42388	1,108,657	1,037,611	281	SAMN18103102	CN107515	756,416	717,865
90	SAMN18102911	CN107873	996,968	936,355	186	SAMN18103007	CN42391	1,031,187	965,016	282	SAMN18103103	CN107521	945,496	898,877
91	SAMN18102912	CN107874	980,293	922,826	187	SAMN18103008	CN42535	1,051,830	984,146	283	SAMN18103104	CN107525	809,729	769,477
92	SAMN18102913	CN107875	972,965	913,898	188	SAMN18103009	CN44031	1,074,837	1,004,345	284	SAMN18103105	CN107535	869,072	825,683
93	SAMN18102914	CN107876	957,968	902,487	189	SAMN18103010	CN45092	976,063	911,713	285	SAMN18103106	CN107538	269,633	255,484
94	SAMN18102915	CN107877	829,811	783,826	190	SAMN18103011	CN52632	929,780	870,382	286	SAMN18103107	CN107540	330,902	315,734
95	SAMN18102916	CN107878	928,516	872,394	191	SAMN18103012	CN107493	935,165	873,579	287	SAMN18103108	CN107541	461,470	439,921
96	SAMN18102917	XCN35382	822,346	773,329	192	SAMN18103013	X1CN35382	1,013,386	947,816	288	SAMN18103109	CN107543	780,742	742,376

289	SAMN18103110	CN30629	393,789	329,357	385	SAMN18103206	CN30318	991,158	875,271	481	SAMN18103302	CN35332	1,259,303	1,146,070
290	SAMN18103111	CN32352	859,171	728,496	386	SAMN18103207	CN30644	902,438	810,382	482	SAMN18103303	CN35341	967,128	877,086
291	SAMN18103112	CN32631	668,302	569,839	387	SAMN18103208	CN31631	1,467,720	1,366,545	483	SAMN18103304	CN39109	1,239,337	1,125,077
292	SAMN18103113	CN32669	898,581	773,597	388	SAMN18103209	CN31719	962,923	866,681	484	SAMN18103305	CN107461	1,084,902	1,001,145
293	SAMN18103114	CN35310	737,852	624,892	389	SAMN18103210	CN31984	1,056,784	953,410	485	SAMN18103306	CN107665	1,069,497	970,125
294	SAMN18103115	CN35311	862,751	740,011	390	SAMN18103211	CN32354	849,606	767,141	486	SAMN18103307	CN107810	931,332	855,393
295	SAMN18103116	CN35344	895,098	772,870	391	SAMN18103212	CN35337	933,276	858,728	487	SAMN18103308	CN107811	1,015,903	914,405
296	SAMN18103117	CN39071	702,305	603,640	392	SAMN18103213	CN35342	1,150,237	1,056,515	488	SAMN18103309	CN107813	1,273,402	1,151,055
297	SAMN18103118	CN39168	852,797	726,588	393	SAMN18103214	CN35383	1,189,190	1,073,838	489	SAMN18103310	CN107814	853,396	772,656
298	SAMN18103119	CN52636	538,164	466,415	394	SAMN18103215	CN36009	1,444,432	1,326,724	490	SAMN18103311	CN107815	820,591	754,917
299	SAMN18103120	CN52644	786,541	654,533	395	SAMN18103216	CN36218	1,334,650	1,232,944	491	SAMN18103312	CN107816	860,495	791,056
300	SAMN18103121	CN52651	900,665	755,165	396	SAMN18103217	CN52628	949,634	864,704	492	SAMN18103313	CN107819	875,822	787,150
301	SAMN18103122	CN52653	630,697	571,212	397	SAMN18103218	CN107473	876,135	800,409	493	SAMN18103314	CN107820	933,304	859,857
302	SAMN18103123	CN107424	875,466	733,456	398	SAMN18103219	CN107512	841,590	765,557	494	SAMN18103315	CN107826	877,851	807,226
303	SAMN18103124	CN107513	706,540	621,698	399	SAMN18103220	CN107592	1,252,539	1,148,616	495	SAMN18103316	CN107830	750,106	686,903
304	SAMN18103125	CN107531	868,564	745,841	400	SAMN18103221	CN107602	922,555	836,456	496	SAMN18103317	CN107832	829,139	757,889
305	SAMN18103126	CN107544	835,802	716,130	401	SAMN18103222	CN107831	818,706	735,710	497	SAMN18103318	CN107836	1,231,237	1,097,309
306	SAMN18103127	CN107545	572,425	531,658	402	SAMN18103223	CN107834	794,329	708,547	498	SAMN18103319	CN107848	914,233	822,292
307	SAMN18103128	CN107546	375,962	333,943	403	SAMN18103224	CN35323	803,930	731,163	499	SAMN18103320	CN107856	1,115,688	1,016,082
308	SAMN18103129	CN107547	489,520	431,342	404	SAMN18103225	CN35333	952,997	866,588	500	SAMN18103321	CN107858	793,495	722,655
309	SAMN18103130	CN107548	421,824	377,197	405	SAMN18103226	CN35334	993,541	902,734	501	SAMN18103322	CN107860	884,176	789,832
310	SAMN18103131	CN107552	736,097	647,581	406	SAMN18103227	CN35343	935,254	848,909	502	SAMN18103323	CN107861	844,582	762,825
311	SAMN18103132	CN107553	654,498	570,639	407	SAMN18103228	CN35354	927,833	845,891	503	SAMN18103324	CN107870	1,001,505	901,150
312	SAMN18103133	CN107554	709,536	602,243	408	SAMN18103229	CN35356	1,386,495	1,281,299	504	SAMN18103325	CN29742	897,906	812,420
313	SAMN18103134	CN107555	936,859	888,361	409	SAMN18103230	CN35357	1,045,931	975,534	505	SAMN18103326	CN29747	1,211,407	1,123,314
314	SAMN18103135	CN107556	1,207,800	1,131,405	410	SAMN18103231	CN35361	991,055	924,631	506	SAMN18103327	CN29749	1,013,837	944,643
315	SAMN18103136	CN107558	1,064,359	1,003,053	411	SAMN18103232	CN35756	994,120	930,995	507	SAMN18103328	CN29750	1,171,551	1,102,718
316	SAMN18103137	CN107560	997,510	944,942	412	SAMN18103233	CN35917	1,017,884	950,045	508	SAMN18103329	CN29790	924,852	860,799
317	SAMN18103138	CN107561	1,065,565	1,007,675	413	SAMN18103234	CN36008	396,080	369,587	509	SAMN18103330	CN29798	1,046,733	978,587
318	SAMN18103139	CN107562	1,167,829	1,100,881	414	SAMN18103235	CN36214	911,236	853,912	510	SAMN18103331	CN32001	876,192	821,919
319	SAMN18103140	CN107563	1,208,851	1,135,105	415	SAMN18103236	CN36216	974,605	909,148	511	SAMN18103332	CN33247	1,069,330	1,001,510
320	SAMN18103141	CN107568	1,196,084	1,127,216	416	SAMN18103237	CN36217	1,171,339	1,097,515	512	SAMN18103333	CN33251	901,947	837,270
321	SAMN18103142	CN107566	1,133,717	1,073,527	417	SAMN18103238	CN36336	1,359,822	1,267,872	513	SAMN18103334	CN33254	1,090,332	1,019,988
322	SAMN18103143	CN107569	1,035,185	979,193	418	SAMN18103239	CN39077	1,185,040	1,105,925	514	SAMN18103335	CN33257	1,116,144	1,041,491
323	SAMN18103144	CN107571	1,146,178	1,085,972	419	SAMN18103240	CN39084	1,120,190	1,046,819	515	SAMN18103336	CN33258	903,408	840,268
324	SAMN18103145	CN107572	956,470	902,879	420	SAMN18103241	CN39086	1,076,100	1,008,756	516	SAMN18103337	CN33260	865,095	803,561
325	SAMN18103146	CN107573	1,196,419	1,136,008	421	SAMN18103242	CN39087	650,290	606,706	517	SAMN18103338	CN33263	950,015	890,895
326	SAMN18103147	CN107574	1,410,167	1,338,035	422	SAMN18103243	CN39096	974,661	914,221	518	SAMN18103339	CN33264	866,125	806,681
327	SAMN18103148	CN107577	927,874	879,952	423	SAMN18103244	CN39099	1,147,717	1,075,185	519	SAMN18103340	CN33266	946,319	879,686
328	SAMN18103149	CN107578	908,696	854,454	424	SAMN18103245	CN39114	1,122,789	1,056,064	520	SAMN18103341	CN33267	1,034,703	970,386
329	SAMN18103150	CN107579	760,526	714,594	425	SAMN18103246	CN39131	1,192,959	1,113,880	521	SAMN18103342	CN33277	1,017,583	951,227
330	SAMN18103151	CN107580	1,161,306	1,103,412	426	SAMN18103247	CN39150	1,618,902	1,521,313	522	SAMN18103343	CN33278	1,156,219	1,078,743
331	SAMN18103152	CN107581	911,884	865,729	427	SAMN18103248	CN39169	1,051,157	981,038	523	SAMN18103344	CN33279	1,029,580	956,410
332	SAMN18103153	CN107582	902,398	855,367	428	SAMN18103249	CN39193	995,627	929,768	524	SAMN18103345	CN35264	917,362	858,151
333	SAMN18103154	CN107583	1,086,979	1,030,407	429	SAMN18103250	CN42536	933,446	872,301	525	SAMN18103346	CN35265	1,036,800	969,185
334	SAMN18103155	CN107585	788,221	739,642	430	SAMN18103251	CN51844	951,012	892,623	526	SAMN18103347	CN35319	950,249	883,503
335	SAMN18103156	CN107588	1,046,265	990,117	431	SAMN18103252	CN51845	971,469	907,456	527	SAMN18103348	CN35330	835,354	781,186
336	SAMN18103157	CN107593	947,791	895,539	432	SAMN18103253	CN107353	1,063,837	995,648	528	SAMN18103349	CN35331	987,470	921,925
337	SAMN18103158	CN107594	848,579	798,984	433	SAMN18103254	CN107385	786,728	730,006	529	SAMN18103350	CN35339	1,200,681	1,109,452
338	SAMN18103159	CN107595	1,092,019	1,026,138	434	SAMN18103255	CN107397	1,135,566	1,057,052	530	SAMN18103351	CN35364	1,004,690	931,793
339	SAMN18103160	CN107596	1,218,501	1,136,059	435	SAMN18103256	CN107408	938,783	871,639	531	SAMN18103352	CN36135	916,495	849,508
340	SAMN18103161	CN107597	1,079,713	1,019,018	436	SAMN18103257	CN107476	1,098,070	1,024,600	532	SAMN18103353	CN36215	935,422	867,077
341	SAMN18103162	CN107599	1,260,987	1,172,356	437	SAMN18103258	CN107480	962,871	893,132	533	SAMN18103354	CN36249	1,271,964	1,192,657
342	SAMN18103163	CN107600	1,142,124	1,062,084	438	SAMN18103259	CN107490	940,009	874,733	534	SAMN18103355	CN39075	868,263	807,362
343	SAMN18103164	CN107603	1,156,064	1,094,789	439	SAMN18103260	CN107492	991,830	925,091	535	SAMN18103356	CN39178	995,552	926,207
344	SAMN18103165	CN107604	698,136	658,498	440	SAMN18103261	CN107494	747,865	697,456	536	SAMN18103357	CN42220	1,278,259	1,193,253
345	SAMN18103166	CN107605	1,460,122	1,383,190	441	SAMN18103262	CN107495	1,279,806	1,188,749	537	SAMN18103358	CN42379	1,178,901	1,096,112
346	SAMN18103167	CN107606	1,134,855	1,067,087	442	SAMN18103263	CN107496	893,923	826,468	538	SAMN18103359	CN42387	926,478	858,064
347	SAMN18103168	CN107608	644,731	606,034	443	SAMN18103264	CN107497	1,102,065	1,027,230	539	SAMN18103360	CN45056	1,117,905	1,035,993
348	SAMN18103169	CN107609	1,139,439	1,073,395	444	SAMN18103265	CN107498	984,669	914,026	540	SAMN18103361	CN46335	885,883	820,715
349	SAMN18103170	CN107610	1,179,304	1,100,565	445	SAMN18103266	CN107510	926,458	861,854	541	SAMN18103362	CN51846	1,158,118	1,078,030
350	SAMN18103171	CN107611	609,116	568,223	446	SAMN18103267	CN107514	917,656	852,041	542	SAMN18103363	CN52740	919,173	857,334
351	SAMN18103172	CN107613	1,170,172	1,107,373	447	SAMN18103268	CN107516	845,565	785,156	543	SAMN18103364	CN52874	966,878	905,903
352	SAMN18103173	CN107615	973,260	914,107	448	SAMN18103269	CN107517	1,172,492	1,092,173	544	SAMN18103365	CN52877	980,838	913,341
353	SAMN18103174	CN107616	1,096,397	1,029,584	449	SAMN18103270	CN107518	1,227,503	1,140,701	545	SAMN18103366	CN107352	962,460	890,909
354	SAMN18103175	CN107617	860,153	813,997	450	SAMN18103271	CN107519	1,066,693	991,765	546	SAMN18103367	CN107359	951,927	880,897
355	SAMN18103176	CN107618	972,291	919,234	451	SAMN18103272	CN107520	862,049	801,671	547	SAMN18103368	CN107360	1,014,653	939,510
356	SAMN18103177	CN107619	729,615	680,122	452	SAMN18103273	CN107522	967,137	896,238	548	SAMN18103369	CN107361	1,185,433	1,108,605
357	SAMN18103178	CN107620	1,239,194	1,169,846	453	SAMN18103274	CN107523	1,014,332	946,715	549	SAMN18103370	CN107370	646,772	602,501
358	SAMN18103179	CN107621	839,445	795,131	454	SAMN18103275	CN107526	1,031,854	959,131	550	SAMN18103371	CN107373	1,108,417	1,036,870
359	SAMN18103180	CN107622	974,752	923,232	455	SAMN18103276	CN107527	950,299	881,595	551	SAMN18103372	CN107375	930,948	860,436
360	SAMN18103181	CN107623	1,086,182	1,020,957	456	SAMN18103277	CN107530	1,082,977	1,011,904	552	SAMN18103373	CN107389	1,297,739	1,221,680
361	SAMN18103182	CN107624	1,098,158	1,047,876	457	SAMN18103278	CN107532	1,170,847	1,096,785	553	SAMN18103374	CN107391	1,000,064	932,767
362	SAMN18103183	CN107625	809,993	772,332	458	SAMN18103279	CN107534	1,091,732	1,022,164	554	SAMN18103375	CN107394	1,094,162	1,021,432
363	SAMN18103184	CN107626	832,842	791,860	459	SAMN18103280	CN107536	1,000,724	935,133	555	SAMN18103376	CN107395	893,753	834,282
364	SAMN18103185	CN107627	964,110	918,282	460	SAMN18103281	CN107542	1,108,342	1,032,612	556	SAMN18103377	CN107403	1,254,282	1,171,592
365	SAMN18103186	CN107628	607,018	570,900	461	SAMN18103282	CN107549	912,708	853,003	557	SAMN18103378	CN107405	1,206,336	1,132,400
366	SAMN18103187	CN107629	879,890	839,498	462	SAMN18103283	CN107550	711,207	666,834	558	SAMN18103379	CN107407	937,052	878,255
367	SAMN18103188	CN107632	866,761	820,193	463	SAMN18103284	CN107557	1,023,608	958,544	559	SAMN18103380	CN107413	989,985	925,812
368	SAMN18103189	CN107634	897,687	851,753	464	SAMN18103285	CN107559	1,180,570	1,108,083	560	SAMN18103381	CN107420	1,275,413	1,191,237
369	SAMN18103190	CN107635	929,524	885,523	465	SAMN18103286	CN107565	1,193,252	1,110,782	561	SAMN18103382	CN107421	1,206,798	1,125,415
370	SAMN18103191	CN107636	576,331	543,013	466	SAMN18103287	CN107567	858,341	800,955	562	SAMN18103383	CN107422	1,346,792	1,267,812
371	SAMN18103192	CN107637	900,605	857,765	467	SAMN18103288	CN107575	410,944	382,150	563	SAMN18103384	CN107425	898,209	848,737
372	SAMN18103193	CN107638	857,701	812,532	468	SAMN18103289	CN107576	828,788	769,889	564	SAMN18103385	CN107436	1,109,232	1,031,029
373	SAMN18103194	CN107639	925,928	876,746	469	SAMN18103290	CN107586	909,032	845,758	565	SAMN18103386	CN107438	1,169,800	1,087,546
374	SAMN18103195	CN107640	973,238	923,818	470	SAMN18103291	CN107587	1,129,685	1,052,957	566	SAMN18103387	CN107439	946,526	875,275
375	SAMN18103196	CN107641	875,717	832,105	471	SAMN18103292	CN107591	972,895	905,503	567	SAMN18103388	CN107443	1,152,234	1,066,436
376	SAMN18103197	CN107642	452,037	425,348	472	SAMN18103293	CN107601	1,248,386	1,165,563	568	SAMN18103389	CN107447	1,213,171	1,136,799
377	SAMN18103198	CN107643	883,895	842,550	473	SAMN18103294	CN107607	754,792	706,783	569	SAMN18103390	CN107451	994,592	924,876
378	SAMN18103199	CN107645	658,173	618,592	474	SAMN18103295	CN107612	952,191	889,841	570	SAMN18103391	CN107459	1,008,991	935,875
379	SAMN18103200	CN107646	872,583	833,682	475	SAMN18103296	CN107614	942,560	886,191	571	SAMN18103392	CN107469	939,854	875,072
380	SAMN18103201	CN107647	997,686	944,573	476	SAMN18103297	CN107631	793,788	740,841	572	SAMN18103393	CN107570	1,071,690	995,891
381	SAMN18103202	CN107648	834,742	790,972	477	SAMN18103298	CN107633	1,073,937	1,004,396	573	SAMN18103394	CN107589	1,051,044	975,074
382	SAMN18103203	CN107649	783,005	746,596	478	SAMN18103299	CN107644	961,379	900,647	574	SAMN18103395	CN107802	884,930	826,797
383	SAMN18103204	CN107651	767,563	732,985	479	SAMN18103300	CN107653	938,385	874,762	575	SAMN18103396	CN107825	1,066,287	991,602
384	SAMN18103205	X2CN35382	560,205	531,977	480	SAMN18103301	X3CN35382	825,744	770,237	576	SAMN18103397	X4CN35382	946,206	876,619

Table S4. The pairwise group genetic distances obtained from AMOVA analysis of 24 countries or groups (in the lower diagonal). The corresponding statistical significances are shown in the upper diagonal. A plus sign shows the significant test at $p<0.05$, while the minus sign indicates a non-significant test at $p>0.05$. See the 24 countries or groups in Table 1.

Group	1	2	3	4	5	6	7	8	9	10	11	12	13	14	15	16	17	18	19	20	21	22	23	24
1		+	+	+	+	+	−	+	+	−	−	−	+	+	−	−	−	−	−	+	−	−	+	+
2	0.026		−	+	+	+	+	+	+	+	−	+	+	+	−	−	−	−	−	+	−	−	+	+
3	0.023	0.018		+	+	+	+	+	−	+	−	−	+	−	−	−	−	−	−	+	−	−	+	+
4	0.050	0.048	0.040		+	+	+	−	−	−	+	+	−	−	−	−	−	−	−	+	−	+	−	+
5	0.075	0.049	0.052	0.071		+	+	+	+	+	+	+	+	+	−	−	−	−	−	+	−	−	+	+
6	0.040	0.041	0.044	0.045	0.092		−	+	−	+	−	−	+	−	−	−	−	−	−	−	−	−	−	+
7	0.026	0.034	0.033	0.050	0.077	0.017		+	−	+	−	−	+	+	−	−	−	−	−	−	−	−	−	+
8	0.069	0.058	0.053	0.030	0.058	0.093	0.076		+	−	+	+	−	+	−	−	−	−	−	+	+	+	+	+
9	0.057	0.053	0.044	0.033	0.076	0.028	0.036	0.064		+	+	−	−	−	−	−	−	−	−	+	−	−	−	+
10	0.027	0.041	0.035	0.024	0.072	0.063	0.046	0.033	0.064		−	+	−	+	−	−	−	−	−	+	+	+	+	+
11	0.013	0.023	0.022	0.042	0.076	0.035	0.023	0.060	0.056	0.024		−	+	+	−	−	−	−	−	+	−	−	+	+
12	0.047	0.060	0.050	0.083	0.102	0.016	0.021	0.128	0.028	0.092	0.051		+	+	−	−	−	−	−	−	−	−	−	+
13	0.087	0.076	0.074	0.010	0.095	0.070	0.071	0.049	0.053	0.046	0.070	0.105		−	−	−	−	−	−	+	−	+	−	+
14	0.091	0.087	0.073	0.057	0.102	0.077	0.106	0.109	0.093	0.126	0.126	0.144	0.079		+	−	+	−	−	+	−	−	−	+
15	0.056	0.053	0.054	0.061	0.056	0.090	0.073	0.047	0.070	0.060	0.051	0.110	0.075	0.180		−	−	−	−	−	−	+	−	+
16	0.031	0.046	0.031	0.024	0.062	0.029	0.021	0.059	0.017	0.043	0.041	0.038	0.031	0.114	0.077		−	−	−	−	−	−	−	+
17	0.091	0.083	0.086	0.120	0.122	0.137	0.107	0.112	0.127	0.099	0.083	0.149	0.151	0.227	0.098	0.115		−	−	−	−	−	+	+
18	0.034	0.027	0.025	0.032	0.069	0.037	0.066	0.073	0.070	0.057	0.054	0.081	0.067	0.042	0.094	0.091	0.091		−	−	−	−	−	+
19	0.098	0.083	0.081	0.061	0.125	0.082	0.127	0.126	0.102	0.126	0.127	0.146	0.089	0.007	0.158	0.130	0.204	-0.023		−	−	−	−	+
20	0.214	0.250	0.238	0.276	0.312	0.150	0.168	0.350	0.218	0.322	0.238	0.145	0.301	0.332	0.357	0.283	0.351	0.249	0.304		−	−	−	+
21	0.125	0.147	0.124	0.131	0.168	0.058	0.083	0.198	0.085	0.220	0.139	0.089	0.157	0.085	0.241	0.134	0.276	0.108	0.108	0.193		−	−	+
22	0.173	0.221	0.201	0.220	0.276	0.073	0.103	0.325	0.144	0.302	0.187	0.074	0.253	0.270	0.403	0.272	0.387	0.178	0.225	-0.015	0.165		−	−
23	0.093	0.108	0.100	0.062	0.130	0.032	0.058	0.140	0.051	0.125	0.101	0.057	0.076	0.086	0.170	0.053	0.220	0.070	0.094	0.217	0.055	0.143		+
24	0.123	0.178	0.170	0.238	0.246	0.198	0.206	0.285	0.261	0.216	0.181	0.225	0.291	0.238	0.254	0.252	0.270	0.162	0.209	0.357	0.300	0.285	0.279	
